# Genetic Evolution Characteristics of Genotype G57 Virus, A Dominant Genotype of H9N2 Avian Influenza Virus

**DOI:** 10.3389/fmicb.2021.633835

**Published:** 2021-03-03

**Authors:** Jinfeng Wang, Xuanjiang Jin, Jingkai Hu, Yifan Wu, Mengmeng Zhang, Xiao Li, Jianglin Chen, Shumin Xie, Jing Liu, Wenbao Qi, Ming Liao, Weixin Jia

**Affiliations:** ^1^National Avian Influenza Para-Reference Laboratory (Guangzhou), College of Veterinary Medicine, South China Agricultural University, Guangzhou, China; ^2^Experimental Animal Center, South China Agricultural University, Guangzhou, China; ^3^Key Laboratory of Zoonosis, Ministry of Agriculture and Rural Affairs, Guangzhou, China; ^4^Guangdong Laboratory for Lingnan Modern Agriculture, Guangzhou, China; ^5^National and Regional Joint Engineering Laboratory for Medicament of Zoonosis Prevention and Control, Guangzhou, China; ^6^Key Laboratory of Zoonoses Prevention and Control of Guangdong Province, Guangzhou, China

**Keywords:** H9N2, avian influenza virus, genotype G57, nucleotide substitution rate, positive selection

## Abstract

This study aimed to investigate the genetic evolution of the H9N2 avian influenza virus (AIV). Whole genome phylogenetic trees were constructed based on 306 H9N2 avian influenza strains collected in China from 2014 to 2019. The results showed that eight gene sequences were clustered separately according to their dominant clades, and a total of 10 genotypes were identified (seven of which were novel types). Among them, G57 genotype was confirmed as the most prevalent genotype with a frequency of 94%. In China, the G57 genotype of H9N2 first emerged in 2007, and then became the most common genotype in 2013. Therefore, the nucleotide substitution rates of G57 genotype in HA and NA genes collected from 2007 to 2019 were estimated, and the positive selection pressure sites in the same data set were measured. Taking 2013 as the boundary, the time period was divided into two periods: 2007–2012 and 2013–2019. From 2007 to 2012, multiple genotypes coexisted and could bear the pressures from both nature and environment; while G57 genotype was still in the adaptation stage, subjected to less selection pressure and in the process of slow evolution. However, from 2013 to 2019, G57 became the dominant genotype, and most of the external pressure reacted on it. Moreover, G57 genotype showed better adaptability than other genotypes. From 2013 to 2019, the nucleotide substitution rates of the HA gene were increased, and the positive selection pressures on HA and NA genes were stronger compared to those from 2007 to 2012. To sum up, the absolutely dominant G57 genotype exhibited a relatively constant genotype frequency and experienced adaptive evolution and natural selection simultaneously during the monitoring period. Therefore, urgent attention and diligent surveillance of H9N2 avian influenza virus are becoming increasingly important.

## Introduction

The H9N2 avian influenza virus (AIV) was originally isolated from turkeys in the United States state of Wisconsin in 1966 (Homme and Easterday, 1970). Since then, the virus has spread worldwide. China is considered as an epidemic center of H9N2 AIV infection (Shortridge et al., 2000). The AIV genome consists of eight distinct RNA segments, which encode more than 10 viral proteins. Previous research has shown that the hemagglutinin (HA) gene of H9N2 diverged into both Eurasian and American lineages (Guan et al., 1999). The Eurasian lineage is further subdivided into four lineages, namely, BJ/94-like, Y280-like, G1-like, and Y439-like (Li et al., 2005; Lu et al., 2005; Xu et al., 2007; Fusaro et al., 2011). BJ/94-like and G1-like are mainly found in China (Xu et al., 2007; Zhang et al., 2008). The F/98-like appeared in 1998 and gradually rose as a major lineage in China (Zhang et al., 2008; Huang et al., 2010).

To further understand the evolution of the HA gene, Li categorized H9N2 from China into 16 phylogenetic groups that represent isolates from 1994 to 2013. HA clades were categorized as a clade number between 0 and 15. Among these clades, 11 of them evolved from the BJ/94-like cluster of viruses (clade 5–15). Apart from BJ/94-like, the HA genes of the other lineages did not undergo extensive variation, G1-like formed a phylogenetic clade designated as number 4 and Y439-like formed a clade marked as number 2 (Li et al., 2017). In the case where two or more AIV subtypes infect a cell, genetic reassortment may allow an exchange of genetic material between them (Gu et al., 2014b). Similarly, H9N2 generate a large number of genotypes by frequent genetic reassortment with the other AIV subtypes (Gu et al., 2017). Li identified 117 genotypes among 730 H9N2 isolated from China between 1994 and 2013, including 45 major genotypes, that continuously occur over this time frame, and 72 transiently occurring genotypes, that appear only once per year. Interestingly, the G57 genotype is the most frequent genotype and the only genotype during 2013 (Li et al., 2017).

The G57 genotype first appeared in chickens in Eastern China during 2007 and has become the dominant genotype across China since 2010. This G57 genotype has undergone continual reassortment of gene segments. The neuraminidase (NA) gene of G57 was derived from the BJ/94-like lineage in 1994. In 1998, the polymerase basic (PB1), polymerase acidic (PA), nucleoprotein (NP), and non-structural (NS) genes were produced in the F/98-like lineage, which led to a severe outbreak in chickens across Eastern China. After 1998, the F/98-like virus reassorted with the A/quail/HongKong/G1/1997-like and A/chicken/Jiangsu/1/2000-like viruses, introducing matrix (M) and HA genes to generate a new variant during 2005. At that time, a genetic segment named polymerase basic 2 (PB2) was transmitted from ducks to chickens, where it formed the predominant G57 genotype in 2007 (Pu et al., 2015). Since 2010, G57 widely circulated in chickens across China, and its six internal genes constitute a relatively stable community to transfer into other novel reassortants. An outbreak caused by a novel H7N9 virus was first reported in China in 2013, which could be a c product from the H9N2 AIV (Pu et al., 2015). During that year, a novel reassortant virus, WZ-Ck-H7N7, was detected in Wenzhou, and a novel virus, H10N8, infected humans in the Jiangxi Province (Lam et al., 2013; Gu et al., 2014a). Interestingly, the internal gene donors of these viruses were presumed to be G57 genotype. Previous research has identified 117 genotypes from a sample of 730 H9N2 strains isolated across China between 1994 and 2013 (Li et al., 2017). However, the genotypic evolution of H9N2 AIV since 2014 is not well established. To better understand the genetic evolution of this virus, 306 H9N2 AIV strains collected in China between 2014 and 2019 were analyzed.

## Materials and Methods

### Virus Isolation

Oropharyngeal and cloacal swab specimens were collected from poultry in the fixed live poultry markets in different urban areas of Guangdong Province from 2017 to 2019. Meanwhile, viral samples were obtained from the Center for Disease Control (CDC, Guangdong Province) and other farms across Shandong and Yunnan provinces. Each sample was prepared in a 1 mL of phosphate buffered saline (PBS) containing 20% glycerol and penicillin-streptomycin mixture (5,000 UI/mL). After centrifugation at 10,000 *g* for 5 min, the supernatant was inoculated into the allantoic cavity of 10-day-old specific pathogen-free embryonated chicken eggs and then incubated at 37°C for 48–72 h. The allantoic fluids were then harvested, and H9N2 strains were identified by a combination of Hemagglutination (HA) test and Hemagglutination inhibition (HI) test (Eisfeld et al., 2014). H9N2 virus sequences containing eight gene segments obtained from multi-regions and multi-hosts in China from 2014 to 2018 were used to screen these samples, which were available from the Global Initiative of Sharing All Influenza Data (GISAID) database^1^.

### RNA Extraction and PCR Amplification

RNA was extracted from hemagglutination-positive allantoic fluid using Total RNA Speed Extraction Kit according to the manufacturer’s instructions (Shanghai Feijie Biotechnology Co., Ltd.). Then, RNA was reverse-transcribed into cDNA using the M-MLV reverse transcriptase (TaKaRa) with Uni12 primer (5′-AGCAAAAGCAGG-3′) for 2 h at 42°C (Hoffmann et al., 2001). After reverse transcription, eight full-length influenza virus genes were amplified using the ExTaq TM DNA polymerase (TaKaRa) with fragment-specific primers. The PCR program was set as follows: initial denaturation at 94°C for 5 min, followed by 33 cycles of 94°C for 50 s, 55°C for 50 s, and 72°C for 1 min and 50 s, and final extension at 4°C for 5 min. Gel purified PCR products of all eight segments of these viruses were directly sequenced at by TSINGKE Co., Ltd. (Guangdong province, China).

### Sequencing and Phylogenetic Analyses

The nucleotide sequences were processed using the Lasergene sequence analysis software based on the National Center for Biotechnology Information (NCBI) virus database^2^. MAFFT version 7.058 was used to align each of the eight gene segments and eliminate the sequences with less than 95% of the expected segment length. Finally, the same sequences in the gene fragment were removed by PhyloSuite. The phylogenetic tree was reconstructed by aligning the open reading frames that correspond to those codes for complete protein. The best-fitting nucleotide substitution model was selected using the Akaike Information Criterion (AIC) as implemented in MrModeltest version 3.2 (Huelsenbeck and Ronquist, 2001). Four independent Markov Chain Monte Carlo (MCMC) analyses were run independently for 200 million generations and sampled every 20,000 generations. The convergence of MCMC was assessed by the effective sample size (ESS) of at least 200 using Tracer version 1.7. Following a burn-in of 10% for each run, the remaining 18,000 trees were reconstructed for creating the Maximum Clade Credibility (MCC) tree (Li et al., 2020). High-quality visualization of the phylogenetics data was performed by the Interactive Tree of Life (iTOL^3^).

### Genotype Analyses

Viral genotypes were classified according to the phylogenetic analysis of a combined set of eight RNA segments. The H9N2 gene sequences were derived from the virus sequences isolated in the laboratory, while other related sequences were obtained from GISAID database. The genotype names were defined based on prior research reports as well as from the results of this study. Previous studies (Li et al., 2017; Jin et al., 2020) had identified 118 genotypes (G01–G118), and the novel genotypes identified in this study were designated by the numbers in chronological order.

### Estimation of Nucleotide Substitution Rates

The rate of nucleotide substitution was calculated by the Bayesian Markov chain Monte Carlo (MCMC) method implemented in the BEAST version 1.8.4. The best-fitting nucleotide substitution model was selected based on the Bayesian information criterion in ModelFinder (Kalyaanamoorthy et al., 2017) through IQ-TREE 1.2.1 (Zhang et al., 2020). This analysis included path sampling (PS) and stepping-stone sampling (SS) to select the best fitting clock models (the strict clock and uncorrelated lognormal relaxed clock) and the most appropriate tree prior (the constant size, exponential growth, and Bayesian skyline coalescent) (Drummond and Rambaut, 2007; Baele et al., 2012; Supplementary Table 1). Specifically, the period of viral isolation was used to calibrate the above molecular clock. Four independent MCMC chains were run simultaneously for 200 million generations, followed by sampling at an each interval for 20,000 generations. Convergence of all parameters and their values were visually verified using Tracer version 1.7 (Rambaut et al., 2018). The uncertainty of each parameter was reported as the value of the 95% Highest Probability Density (HPD).

### Analysis of Natural Selection at the Molecular Level

Positive Darwinian selection was carried out using the Datamonkey web server^4^ (Pond and Frost, 2005). The fixed effects likelihood (FEL), mixed effects model of evolution (MEME), and fast unconstrained Bayesian approximation for inferring selection (FUBAR) were proposed to estimate the selection pressure (Hu et al., 2017). The codon sites under positive selection were calculated by at least two of the above three methods in order to examine the validity of the obtained data (Murrell et al., 2012, 2013).

## Results

### Isolation of Viral Samples

**FIGURE 1:**
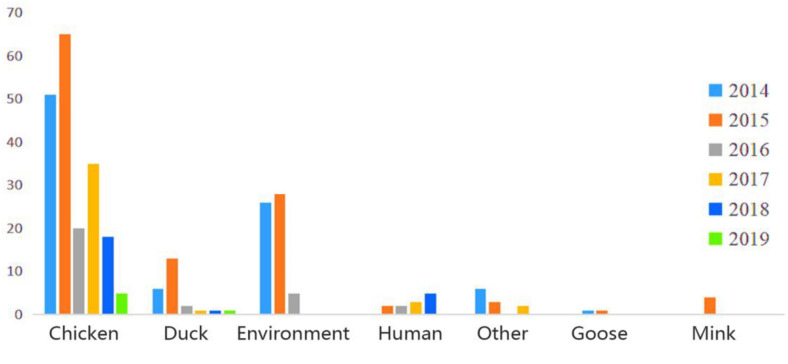
Host and time information of H9N2 AIV strains isolated from China during 2014–2019.

**FIGURE 2:**
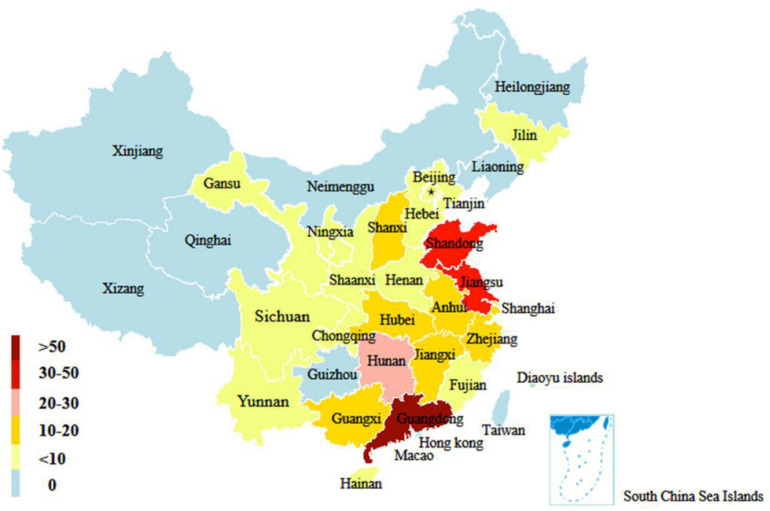
Geographical distribution of H9N2 AIV strains in China from 2014 to 2019. The number of color legend represents the number of virus strains isolated in the corresponding color-marked provinces. For example, more than 50 H9N2 viruses were isolated in Guangdong from 2014 to 2019.

In this study, a total of 306 H9N2 AIV strains were sampled. Of them, 33 were isolated between 2017 and 2019 and the remaining 273 strains were screened from the GISAID database between 2014 and 2018 (Supplementary Table 2). The distribution of H9N2 AIV strains by host was as follows: 220 strains were from poultry (e.g., chickens, ducks, and geese); 59 strains from environmental samples; 16 strains were from humans and other mammals (e.g., mink); and the remaining were from other miscellaneous animal species. The distribution of H9N2 AIV by year included: 89 strains isolated in 2014; 116 strains in 2015; 30 strains in 2016; 41 strains in 2017; 24 strains in 2018; and six strains in 2019. The sequence data of H9N2 AIV covered 25 regions in China (Figures 1, 2).

### Phylogenetic Analyses of the Surface Genes of H9N2 AIV

**FIGURE 3:**
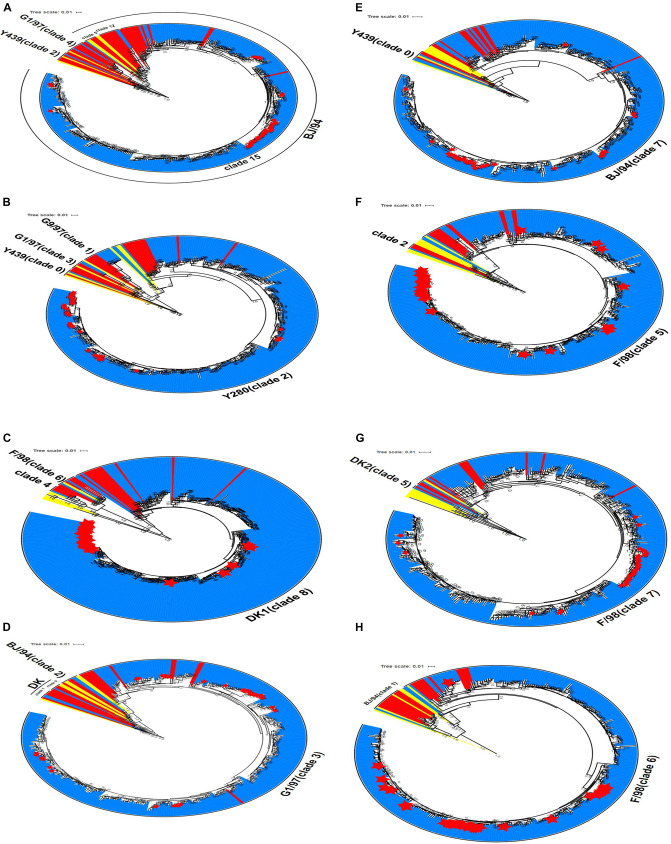
Phylogenetic analyses of eight gene sequences of H9N2 AIV. **(A)** HA gene, **(B)** NA gene **(C)** PB2 gene, **(D)** M gene, **(E)** NS gene, **(F)** PB1 gene, **(G)** PA gene, and **(H)** NP gene. Red denotes the reference sequences, yellow the classical sequences, blue the analysis sequences, and the red star corresponds to the laboratory isolates used in this study. The scale bar shows the number of nucleotide substitutions per site. The cluster assignments for H9N2 are based on Li and Jin et al.

Phylogenetic analysis was performed for the 306 H9N2 strains collected in China between 2014 and 2019. The results showed that the HA gene sequences of H9N2 AIV were clustered into different clades (the number of viral strains is in parentheses): clade 2 (1); clade 4 (2); clade 5 (1); clade 12 (2); and clade 15 (300). The HA gene sequences of the isolates in clade 15 were evolved from the BJ/94-like lineage (Figure 3A). Similarly, the NA genes of H9N2 AIV were clustered into four independent clades: clade 0 (1); clade 1 (7); clade 2 (296); and clade 3 (2). The majority of strains in clade 2, including all laboratory isolates, were originated from the Y439-like lineage (Figure 3B).

### Phylogenetic Analysis of the Internal Genes of H9N2 AIV

The evolutionary origin of the internal genes of H9N2 AIV was more extensive than that of its surface genes. Phylogenetic groups of the internal H9N2 genes were based on the previously identified host origins, genetic relatedness with other strains, geographical location of isolation, or by viral subtypes, such as DK1, G1, and Korean lineage (Chuvakova et al., 1985; Xu et al., 2007; Sun et al., 2010). In this study, instead numerical values were used to denote each clade. PB2 gene, which originated from DKI virus, was predominantly found in clades 4, 6, and 8, and 303 H9N2 virus samples were assigned into clade 8. The M gene was distributed among clades 0, 1, 2, and 3, and 302 strains formed clade 3, which has previously been characterized by the G1-like lineage. For the NS gene, two strains were classified into clade 0, and the remaining 304 strains were belonged to clade 7, which consisted of the BJ/94-like NS segment. The phylogenetic trees of PB1, PA, and NP genes showed that only one strain (A/duck/Wuhan/WHYF05/2014) was grouped in other clades, while the remaining strains were clustered among clades 5, 6, and 7, which putatively evolved from the SF/98-like lineage. From 2017 to 2019, the six internal genes of H9N2 virus isolated in the laboratory were clustered to the dominant clades in the phylogenetic tree, which exhibited consistency with the epidemic characteristics of other local strains (Figures 3C–H).

Altogether, our findings revealed that greater than 98% of the six internal genes were clustered into their expected clades in the phylogenetic trees. The six internal gene fragments of all viral strains could be inherited stably, indicating that the combination of the internal genes is more conducive to the stable existence of H9N2 AIV in nature.

### Genotyping

**FIGURE 4:**
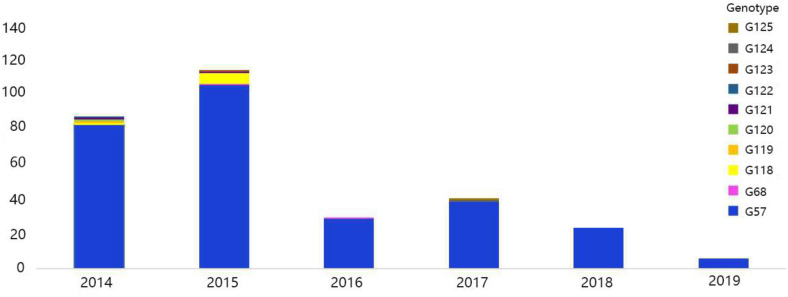
Genotype diversity of H9N2 AIV strains in China from 2014 to 2019. The color bar represents the genotype of the isolated virus, and the ordinate represents the count of genotypes.

**FIGURE 5:**
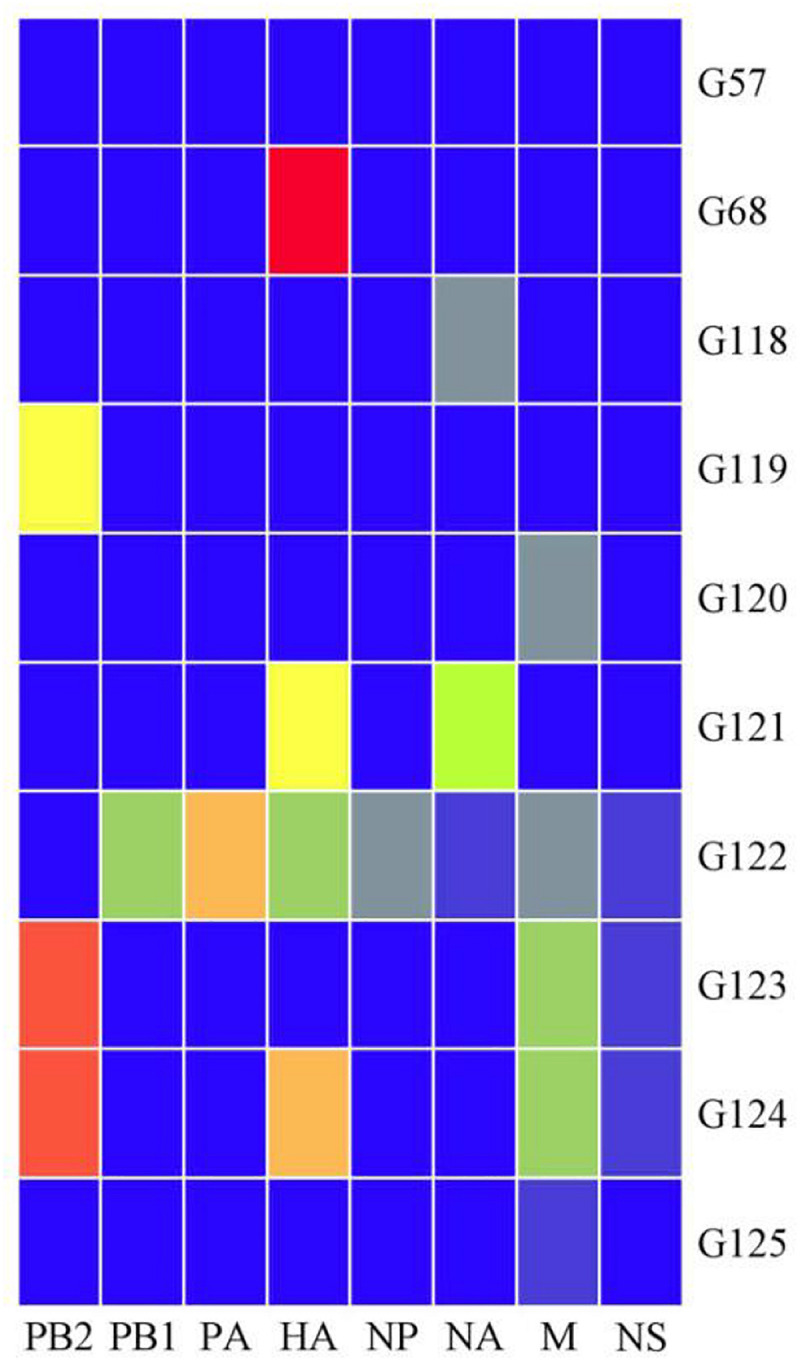
Ten genotypes of H9N2 AIV isolated in China from 2014 to 2019. Eight gene fragments are marked at the top of each strip. Blue indicates the clade of each gene segment of the G57 genotype, and other colors indicate that the clade is different from the clade of the same gene segment in which the G57 genotype is located.

The viral genotypes were identified based on the phylogenetic analysis of eight genes among 306 strains. A total of 10 H9N2 AIV genotypes were identified, including seven novel genotypes, which had not been reported previously. They were assigned with the following names as follows: G119, G120, G121, G122, G123, G124, and G125. Among all genotypes, G57, G68, G118, and G121 were the major genotypes and appeared continually over the study period; while G119, G120, G122, G123, G124, and G125 genotypes were considered as transient. In total, 289 H9N2 strains harbored G57 genotype (94% of all H9N2 types). At expected, the genotypes of laboratory isolates from 2017 to 2019 were shown to harbor G57 genotype, while other genotypes were as follows: seven strains of G118, two strains of G68, two strains of G121, and one strain of each following genotype (G119, G120, G122, G123, G124, and G125) (Figure 4 and Supplementary Table 3). When different types of influenza viruses infect a cell, then reassortment may occur along with the potential for new genotypes. Compared with the G57, G68 was only different in the clade of the HA gene, and G118 was only changed in the clade of the NA gene. The G119 differed only in the clade where the PB2 gene was located, while G120 and G125 differed only in the clade where the M gene was located (Figure 5 and Supplementary Table 3).

### Evolutionary Rate of the G57 Genotype

Given that G57 is the most common genotype among our samples, we further analyzed its genotypic evolution across different regions in China. The G57 genotype of H9N2 first appeared in China in 2007, and it has become the most common genotype before 2013. It should be pointed out that G57 genotype has always been the most dominant genotype in China over the last decade. Therefore, it is proposed that the frequencies of H9N2 genotypes may not change in a substantial way. According to the emergence and evolution of G57 genotype, we intimately divided the H9N2 isolates into two time periods: 2007–2012 (time period 1) and 2013–2019 (time period 2). The nucleotide substitution rates of HA and NA genes during the time period one ranged from 3.38E^–3^ to 6.11E^–3^ and from 3.24E^–3^ to 7.05E^–3^, respectively (Supplementary Table 4). For the time period 2, the nucleotide substitution rates of HA and NA genes ranged from 5.08E^–3^ to 6.74E^–3^ and from 5.09E^–3^ to 6.54E^–3^, respectively (Supplementary Table 4). Notably, the nucleotide substitution rates of HA and NA genes in the second time period were higher than those in the first time period.

### Positive Darwinian Selection of the G57 Genotype

Furthermore, we measured the positive selection of G57 genotype in the two time periods. As shown in Tables 1, 2, there were significant differences in the positive selection of G57 genotype between these two time periods. The HA and NA genes were under relatively strong positive selection in the second time period. There were 11 positive selected sites in the HA gene across the second time period, while only five in the first time period. As for the NA gene, these differences were more pronounced in six codon sites. Specifically, the positive selection was observed in the second time period as compared to only one codon site of this kind detected in the first time period (Supplementary Table 5). In order to adapt in different living environments, H9N2 AIV has acquired different amino acid mutation sites that are beneficial for their survival under natural selection in different periods.

**TABLE 1:** Positively selected codon sites in the HA gene of the G57 genotype.

Period	Number of positively selected sites	Positively selected codon sites
2007–2012	5	3, 4, 13, 145, and 198
2013–2019	11	4, 5, 13, 17, 66, 87, 88, 145, 168,198, and 353

**TABLE 2:** Positively selected codon sites in the NA gene of the G57 genotype.

Period	Number of positively selected sites	Positively selected codon sites
2007–2012	1	9
2013–2019	6	3, 8, 31, 208, 266, and 369

## Discussion

H9N2 AIV has spread around the world, and often co-infects poultry with other pathogens, causing egg production to decline and a high death rate in the poultry population. This may lead to high economic losses (Sun et al., 2010; Shanmuganatham et al., 2013). Prior evolutionary research has focused on H9N2 AIV in China from 1994 to 2013 (Li et al., 2017). We expanded this study by analyzing the H9N2 strains isolated from 2014 to 2019. It should be pointed out that our strain information only includes the host, time, and geographical distribution, but lacks of epidemiological data. In the future, we will consider the spatial and temporal evolution of H9N2 virus. We observed that 99% of the H9N2 AIVs isolated from 2014 to 2019 were distributed in the BJ/94-like lineage, suggesting that BJ/94-like has emerged as the main lineage of H9N2 in China. In addition, 98% of the eight gene fragments were clustered in the same clade in the phylogenetic trees, which consisted of G57 genotype. After two decades of reassortment and mutation in poultry populations, G57 emerged as the most common genotype (94%) in H9N2 AIV.

In China, to prevent chicken from H9N2 AIV infection, inactivated vaccines have been widely used in chicken farm. At present, the A/Chicken/Guangdong/SS/94, A/Chicken/Shandong/6/96 and A/Chicken/Shanghai/F/98 H9N2 subtype strains, which belong to Y280-like lineage, have been employed as seed viruses for the production of inactivated vaccines in China (Xu et al., 2018). However, H9N2 AIVs are often isolated in immunized chickens. In 2010–2013, G57 virus has likely undergone antigenic shift along with adaptation to the environment, which in turns facilitates the virus to escape from host immunity and causes a widespread outbreak (Pu et al., 2015). It has also been reported that the internal gene cassette of G57 is a stable arrangement (Liu et al., 2014). Through reverse genetic methods, Hao et al. (2017) have demonstrated that the internal genes of G57 and the external genes of H5 virus are compatible with one another, and that the reassortant H5 viruses are better adapted to infect poultry and mammals. In 1997, a case of cross-species transmission of H5N1 was reported in Hong Kong, China. The reassortant H5N1 virus that infected humans acquired its internal genes from H9N2, leading to antigenic shift and potential to infect other host species (Wu et al., 2017). During 2013, the first human outbreak of H7N9 occurred in China, which has led to five epidemic waves, 1,568 infected individuals, and 616 deaths. Phylogenetic analysis showed that the six internal genes of H7N9 are constituted of the G57 genotype (Pu et al., 2015). Thus, it is speculated that G57 can adapt to different environments and induce an epidemic by generating new subtypes *via* reassortment. We should pay close attention to the evolutionary characteristics of G57 genotype.

The G57 genotype of H9N2 is predominantly found in China and has not changed in the last 10 years. It is proposed that G57 has reached a relative stasis at the genotypic level. However, AIV is a type of virus with rapid mutation and evolution. Therefore, we performed evolutionary rate analysis and positive selection site analysis of G57 genotype. It was observed that the nucleotide substitution rates of HA gene were higher in 2013–2019 than in 2007–2012, and those of NA gene did not change significantly. In addition, the positive selection pressures on HA and NA genes in 2013–2019 were stronger compared to 2007–2012. Regrettably, this single estimation might neglect the evolutionary background and potential phylogenetic relationship between the sequences in these two time periods.

Antigenic drift and transformation are the main factors in AIV evolution and positive selection is one of the main factors that affect the evolutionary trajectory of AIV (Bush et al., 1999). Nucleotide substitution rate is an important index to evaluate the evolution of AIVs, but there are confounding factors that make it difficult to determine the result. Previous studies have shown that poor vaccine procedures, competition among different branches of the same AIV subtype, gene reassortment, and gene recombination can lead to an increase in nucleotide substitution rates (García et al., 1997; Lee et al., 2004; Bahl et al., 2009; Cattoli et al., 2011). The abnormal changes in nucleotide substitution rate caused by gene reassortment may warn the emergence of new influenza virus (Cox and Subbarao, 2000; Nelson and Holmes, 2007). A previous study in our lab on H9N2 in Guangdong Province has found that the emergence of novel genotypes may also lead to an increase in the nucleotide substitution rate (Jin et al., 2020). Our study showed that novel genotypes appeared in 2014, 2015, and 2017, which could be a potential reason why the nucleotide substitution rates of HA and NA genes from 2013 through 2019 were higher than those from 2007 to 2012. We also speculated that positive natural selection may affect the evolution of G57 in H9 virus. Multiple genotypes of H9N2 coexisted during the 2007–2012 period, bearing the natural and environmental pressure of the outside world. Thus, H9N2 virus may be subjected to less selection pressure. Moreover, at this time, the G57 genotype has not formed the absolutely dominant genotype, and it is in the process of continuous adaptation and slow evolution. Yet from 2013 to 2019, G57 was the most common genotype, and the external selection pressure was all concentrated on this genotype, which led to a strong positive selection pressure in the second period. However, H9N2 AIV with G57 genotype gradually adapted to the current living environment and was in a rapid evolutionary process. We only considered positive selection to focus on mutations that affect the suitability of influenza viruses. Previous research has demonstrate that positive selection on the HA gene of H3N2 may improve the viability of the virus (Bush et al., 1999). H5N1 AIV can undergo positive selection to expand its spread. Under the pressure of positive selection, different mutations are found in a given viral population, which can enhance fitness and produce new viruses that are more suitable for the survival of the whole population.

In summary, G57 genotype, as the absolutely dominant genotype of H9N2 AIV from 2014 to 2019, has undergone intense evolution and adaptive selection. Therefore, urgent attention and diligent surveillance of H9N2 AIV are becoming increasingly essential.

## Data Availability Statement

The raw data supporting the conclusions of this article will be made available by the authors, without undue reservation.

## Author Contributions

All authors listed have made a substantial, direct and intellectual contribution to the work, and approved it for publication.

## Conflict of Interest

The authors declare that the research was conducted in the absence of any commercial or financial relationships that could be construed as a potential conflict of interest.

## Footnotes

1 https://platform.gisaid.org

2 https://www.ncbi.nlm.nih.gov/)

3 https://itol.embl.de/

4 http://www.datamonkey.org)
