# Supplementary material for: Genetic Evolution Characteristics of Genotype G57 Virus, A Dominant Genotype of H9N2 Avian Influenza Virus
Source: Front Microbiol. 2021 Mar 3;12:633835. doi: 10.3389/fmicb.2021.633835 (PMC7965968; doi:10.3389/fmicb.2021.633835)
Supplement: Supplementary file 1 [file Table_1.docx]

**Table S1** The information of 273 H9N2 avian influenza viruses from 2014 to 2018 screened from GISAID database in this study.

| Name | Isolate ID |
| --- | --- |
| A/chicken/Guangdong/04.15SZBAXQ005/2015 | EPI_ISL_199448 |
| A/chicken/Guangdong/04.15SZBAXQ025/2015 | EPI_ISL_199447 |
| A/chicken/Guangdong/04.22DGCP100-O/2015 | EPI_ISL_199282 |
| A/chicken/Guangdong/04.22DGCP101-O/2015 | EPI_ISL_199274 |
| A/chicken/Guangdong/04.22DGCP102-O/2015 | EPI_ISL_199228 |
| A/chicken/Guangdong/04.23DGQTXC191-O/2015 | EPI_ISL_199270 |
| A/chicken/Guangdong/04.23DGQTXC191-P/2015 | EPI_ISL_199225 |
| A/chicken/Guangdong/04.23DGQTXC194-P/2015 | EPI_ISL_199226 |
| A/Anhui/Lujiang/39/2018 | EPI_ISL_330737 |
| A/Anser/fabalis/Anhui/L139/2014 | EPI_ISL_216875 |
| A/Beijing/1/2016 | EPI_ISL_285481 |
| A/Beijing/1/2017 | EPI_ISL_285482 |
| A/chicken/Anhui/03/01/FY001/O/2015 | EPI_ISL_198844 |
| A/chicken/Anhui/AH120/2015 | EPI_ISL_235796 |
| A/chicken/Anhui/AH326/2016 | EPI_ISL_283456 |
| A/chicken/Anhui/AH329/2016 | EPI_ISL_284664 |
| A/chicken/Anhui/AH450/2017 | EPI_ISL_327810 |
| A/chicken/Anhui/AH480/2017 | EPI_ISL_327813 |
| A/chicken/Anhui/LH66/2017 | EPI_ISL_327811 |
| A/chicken/Anhui/LH99/2017 | EPI_ISL_327802 |
| A/chicken/Anhui/WB/2014 | EPI_ISL_294491 |
| A/chicken/China/ShangDong/2018 | EPI_ISL_368393 |
| A/chicken/Daye/DY0602/2017 | EPI_ISL_281313 |
| A/chicken/Dongguan/1674/2014 | EPI_ISL_176412 |
| A/chicken/Fujian/S1XA35/2017 | EPI_ISL_284690 |
| A/chicken/Fujian/SD037/2017 | EPI_ISL_284688 |
| A/chicken/Fujian/SD056/2017 | EPI_ISL_284687 |
| A/chicken/Fujian/SD070/2017 | EPI_ISL_284689 |
| A/chicken/Fujian/SIC16/2014 | EPI_ISL_234445 |
| A/chicken/Ganzhou/GZ126/2016 | EPI_ISL_252840 |
| A/chicken/Ganzhou/GZ140/2016 | EPI_ISL_252839 |
| A/chicken/Ganzhou/GZ86/2016 | EPI_ISL_252835 |
| A/chicken/Guangdong/835/2016 | EPI_ISL_305614 |
| A/chicken/Guangdong/GD1601/2016 | EPI_ISL_284667 |
| A/chicken/Guangdong/SIC17/2014 | EPI_ISL_234432 |
| A/chicken/Guangdong/SIC18/2014 | EPI_ISL_234431 |
| A/chicken/Guangdong/SIC23/2014 | EPI_ISL_234460 |
| A/chicken/Guangdong/SIC28/2014 | EPI_ISL_234459 |
| A/chicken/Guangdong/SIC29/2014 | EPI_ISL_234462 |
| A/chicken/Wuxi/6468/2015 | EPI_ISL_277099 |
| A/chicken/Wuxi/6657/2015 | EPI_ISL_277106 |
| A/chicken/Wuxi/6688/2015 | EPI_ISL_277109 |
| A/chicken/Wuxi/7109/2015 | EPI_ISL_277113 |
| A/chicken/Wuxi/8501/2016 | EPI_ISL_277120 |
| A/chicken/Wuxi/SC4315/2015 | EPI_ISL_223200 |
| A/chicken/Wuxi7723/2016 | EPI_ISL_277119 |
| A/chicken/Xiamen/10/2015 | EPI_ISL_285375 |
| A/chicken/Xuancheng/01/2018 | EPI_ISL_379404 |
| A/chicken/Xuzhou/XZ270/2016 | EPI_ISL_284665 |
| A/chicken/Yunan/07/13/DQDBS051/2015 | EPI_ISL_200830 |
| A/chicken/Yunnan/03/15/DQWGH005/Z/O/2015 | EPI_ISL_199088 |
| A/chicken/Yunnan/03/15/DQXYL0029/O/2015 | EPI_ISL_198879 |
| A/chicken/Yunnan/03/16/DQJT0070/O/2015 | EPI_ISL_201383 |
| A/chicken/Yunnan/03/16/DQJT0071/O/2015 | EPI_ISL_201384 |
| A/chicken/Yunnan/03/16/DQJT062/O/2015 | EPI_ISL_199108 |
| A/chicken/Yunnan/03/16/DQXYL0031/O/2015 | EPI_ISL_199098 |
| A/chicken/Zhejiang/221/2016 | EPI_ISL_253035 |
| A/chicken/Zhejiang/3C34/2014 | EPI_ISL_203714 |
| A/chicken/Zhejiang/727063/2014 | EPI_ISL_203717 |
| A/chicken/Zhejiang/727192/2014 | EPI_ISL_203718 |
| A/chicken/Zhejiang/727198/2014 | EPI_ISL_203719 |
| A/chicken/Zhejiang/77082/2014 | EPI_ISL_203716 |
| A/chicken/Zhejiang/925060/2014 | EPI_ISL_203721 |
| A/chicken/Zhejiang/925117/2014 | EPI_ISL_203722 |
| A/chicken/Zhejiang/925122/2014 | EPI_ISL_203723 |
| A/chicken/Zhejiang/925159/2014 | EPI_ISL_234430 |
| A/chicken/Zhejiang/SIC30/2014 | EPI_ISL_234430 |
| A/chicken/Zhejiang/SIC32/2014 | EPI_ISL_234436 |
| A/chicken/Zhejiang/SIC40/2015 | EPI_ISL_234464 |
| A/chicken/Zhejiang/TL27/2014 | EPI_ISL_272729 |
| A/dove/Guangxi/96B8/2014 | EPI_ISL_278464 |
| A/duck/Anhui/AQ14/2014 | EPI_ISL_272727 |
| A/duck/Ganzhou/GZ188/2016 | EPI_ISL_252836 |
| A/duck/Guangdong/03/26/DGCP101/O/2015 | EPI_ISL_199346 |
| A/duck/Guangdong/03/26/DGCP115/O/2015 | EPI_ISL_199177 |
| A/duck/Guangdong/222/2015 | EPI_ISL_305615 |
| A/duck/Guangdong/A9/2016 | EPI_ISL_305608 |
| A/duck/Hubei/03/06//WHWTZ0140/P/2015 | EPI_ISL_199016 |
| A/chicken/Guangdong/SIC31/2014 | EPI_ISL_234428 |
| A/chicken/Guangdong/SIC37/2015 | EPI_ISL_234461 |
| A/chicken/Guangdong/SIC38/2015 | EPI_ISL_234457 |
| A/chicken/Guangdong/SIC41/2015 | EPI_ISL_234463 |
| A/chicken/Guangxi/C1228/2015 | EPI_ISL_329541 |
| A/chicken/Guangxi/C227/2015 | EPI_ISL_329540 |
| A/chicken/Guangxi/SIC19/2014 | EPI_ISL_234434 |
| A/chicken/Guangxi/SIC20/2014 | EPI_ISL_234433 |
| A/chicken/Guangxi/SIC22/2014 | EPI_ISL_234441 |
| A/chicken/Hainan/SIC33/2014 | EPI_ISL_234466 |
| A/chicken/Henan/815/2016 | EPI_ISL_307920 |
| A/chicken/Huaian/HA9/2016 | EPI_ISL_284663 |
| A/chicken/Hubei/01/2015 | EPI_ISL_279423 |
| A/chicken/Hubei/03/06//WHWTZ0068/P/2015 | EPI_ISL_199023 |
| A/chicken/Hubei/03/06//WHWTZ0088/P/2015 | EPI_ISL_199025 |
| A/chicken/Hubei/03/06/WHWTZ0048/P/2015 | EPI_ISL_199020 |
| A/chicken/Hubei/2014 | EPI_ISL_191490 |
| A/chicken/Hubei/ZYSJF15/2016 | EPI_ISL_252824 |
| A/chicken/Hunan/04/14/YYGK501/O/2015 | EPI_ISL_199125 |
| A/chicken/Hunan/04/14/YYGK506/O/2015 | EPI_ISL_199126 |
| A/chicken/Hunan/04/14/YYGK507/O/2015 | EPI_ISL_199127 |
| A/chicken/Hunan/04/14/YYGK563/P/2015 | EPI_ISL_199129 |
| A/chicken/Hunan/04/22/LDDX046/O/2015 | EPI_ISL_199039 |
| A/chicken/Hunan/04/22/LDDX069/O/2015 | EPI_ISL_199040 |
| A/chicken/Hunan/12/17//YYFQH0015/O/2014 | EPI_ISL_198834 |
| A/chicken/Hunan/YYFQH689/O/2015 | EPI_ISL_199134 |
| A/chicken/Jiangsu/02/06/NJLC042/O/2015 | EPI_ISL_199388 |
| A/chicken/Jiangsu/02/06/NJLC068/O/2015 | EPI_ISL_199387 |
| A/chicken/Jiangsu/03/06/WXBT054/O/2015 | EPI_ISL_199184 |
| A/chicken/Jiangsu/12/30/WZNHQ031/P/2014 | EPI_ISL_199238 |
| A/chicken/Jiangsu/JS4539/2014 | EPI_ISL_235798 |
| A/chicken/Jiangsu/JT138/2016 | EPI_ISL_372494 |
| A/chicken/Jiangsu/JT141/2016 | EPI_ISL_372495 |
| A/chicken/Jiangsu/JT154/2016 | EPI_ISL_372496 |
| A/chicken/Jiangsu/LY2/2017 | EPI_ISL_327804 |
| A/chicken/Jiangsu/SIC42/2015 | EPI_ISL_234467 |
| A/chicken/Jiangsu/TM306/2017 | EPI_ISL_327803 |
| A/chicken/Jiangsu/TM314/2017 | EPI_ISL_327806 |
| A/chicken/Jiangsu/TM315/2017 | EPI_ISL_327807 |
| A/chicken/Jiangsu/TM71/2014 | EPI_ISL_294490 |
| A/chicken/Jiangsu/WJ179/2015 | EPI_ISL_372500 |
| A/chicken/Jiangxi/04/01/NCDTZ0261/P/2015 | EPI_ISL_198931 |
| A/chicken/Jiangxi/04/01/NCDZT0055/O/2015 | EPI_ISL_198983 |
| A/chicken/Jiangxi/04/01/NCDZT0103/O/2015 | EPI_ISL_198751 |
| A/duck/Hunan/01/16//YYGK225/P/2014 | EPI_ISL_198787 |
| A/duck/Hunan/02/26/YYGK250/P/2014 | EPI_ISL_198790 |
| A/duck/Hunan/04/14//YYXS0888/2/P/2015 | EPI_ISL_198877 |
| A/duck/Hunan/04/14/YYGK443/P/2015 | EPI_ISL_199118 |
| A/duck/Hunan/04/14/YYGK901/O/2015 | EPI_ISL_199141 |
| A/duck/Hunan/4/14//YYGK0453/2/O/2015 | EPI_ISL_198859 |
| A/duck/Jiangxi/05/07//NCJD0030A/O/2015 | EPI_ISL_198936 |
| A/duck/ShanDong/JN1/2015 | EPI_ISL_245338 |
| A/duck/Wenzhou/YHQL64/2014 | EPI_ISL_205130 |
| A/duck/Wuhan/WHYF05/2014 | EPI_ISL_205133 |
| A/duck/Wuhan/WHYF14/2014 | EPI_ISL_205134 |
| A/duck/Wuxi/5083/2015 | EPI_ISL_277058 |
| A/duck/Wuxi/6663/2015 | EPI_ISL_277108 |
| A/duck/Yunnan/03/16/DQXYL007/Z/O/2015 | EPI_ISL_199092 |
| A/Environment/Anhui/09183/2014 | EPI_ISL_328594 |
| A/Environment/Anhui/33231/2015 | EPI_ISL_329999 |
| A/environment/Beijing/1/2016 | EPI_ISL_285487 |
| A/environment/Beijing/2/2016 | EPI_ISL_285484 |
| A/environment/Beijing/5/2016 | EPI_ISL_285485 |
| A/Environment/Changzhou/cz96/2014 | EPI_ISL_170232 |
| A/Environment/Chongqing/74343/2014 | EPI_ISL_328597 |
| A/Environment/Fujian/07241/2014 | EPI_ISL_328592 |
| A/Environment/Fujian/85145/2014 | EPI_ISL_328600 |
| A/Environment/Gansu/99800/2014 | EPI_ISL_329994 |
| A/environment/Guangdong/03/27/DGQTSJ042/2015 | EPI_ISL_199396 |
| A/environment/Guangdong/04/22/DGCPLB005/2015 | EPI_ISL_199218 |
| A/Environment/Guangdong/21115/2015 | EPI_ISL_329996 |
| A/Environment/Guangdong/34438/2015 | EPI_ISL_330003 |
| A/Environment/Guangdong/38697/2015 | EPI_ISL_330004 |
| A/Environment/Guangdong/72907/2014 | EPI_ISL_328598 |
| A/Environment/Guangdong/98640/2014 | EPI_ISL_328603 |
| A/Environment/Guangxi/32197/2014 | EPI_ISL_329995 |
| A/Environment/Guangxi/47379/2014 | EPI_ISL_328596 |
| A/Environment/Henan/98615/2014 | EPI_ISL_328599 |
| A/Environment/Hunan/00824/2014 | EPI_ISL_328602 |
| A/environment/Hunan/04/14/YYGK388/2015 | EPI_ISL_199111 |
| A/environment/Hunan/04/14/YYGK400/2015 | EPI_ISL_199114 |
| A/environment/Hunan/26018/2014 | EPI_ISL_179665 |
| A/environment/Hunan/28176/2014 | EPI_ISL_179664 |
| A/environment/Hunan/28184/2014 | EPI_ISL_179663 |
| A/Environment/Hunan/32371/2015 | EPI_ISL_330001 |
| A/Environment/Hunan/32438/2015 | EPI_ISL_330000 |
| A/Environment/Hunan/39658/2015 | EPI_ISL_330006 |
| A/Environment/Hunan/39729/2015 | EPI_ISL_330005 |
| A/chicken/Jiangxi/04/01/NCJD0106/O/2015 | EPI_ISL_198914 |
| A/chicken/Jiangxi/05/06/NCDZT0077B/P/2015 | EPI_ISL_198995 |
| A/chicken/Jiangxi/1202/2014 | EPI_ISL_173535 |
| A/chicken/Jilin/04/04/SY001/O/2015 | EPI_ISL_198778 |
| A/chicken/Jilin/04/05/CCCJ006/2015 | EPI_ISL_198864 |
| A/Chicken/Jilin/13200/2014 | EPI_ISL_161678 |
| A/Chicken/Jilin/13204/2014 | EPI_ISL_161677 |
| A/chicken/Jilin/SD001/2014 | EPI_ISL_168013 |
| A/chicken/Jingmen/JM0305/2017 | EPI_ISL_281312 |
| A/chicken/Qingdao/003/2014 | EPI_ISL_198124 |
| A/chicken/Qingdao/008/2014 | EPI_ISL_198129 |
| A/chicken/Qingdao/009/2014 | EPI_ISL_198130 |
| A/chicken/Qingdao/013/2014 | EPI_ISL_198134 |
| A/chicken/Qingdao/015/2014 | EPI_ISL_198136 |
| A/chicken/Qingdao/017/2014 | EPI_ISL_198138 |
| A/chicken/Qingdao/020/2014 | EPI_ISL_198141 |
| A/chicken/Qingyuan/zd201602/2016 | EPI_ISL_378891 |
| A/chicken/Shandong/1167/2015 | EPI_ISL_195303 |
| A/chicken/ShanDong/210WZ/2017 | EPI_ISL_281315 |
| A/chicken/ShanDong/306SZ/2017 | EPI_ISL_281318 |
| A/chicken/ShanDong/321ZL/2017 | EPI_ISL_281317 |
| A/chicken/ShanDong/413ZDM/2017 | EPI_ISL_281320 |
| A/chicken/Shandong/LY1/2017 | EPI_ISL_309173 |
| A/chicken/Shandong/SIC24/2014 | EPI_ISL_234450 |
| A/chicken/Shandong/SIC25/2014 | EPI_ISL_234451 |
| A/chicken/Shandong/SIC26/2014 | EPI_ISL_234456 |
| A/chicken/Shandong/SIC34/2014 | EPI_ISL_234453 |
| A/chicken/Shandong/SIC35/2014 | EPI_ISL_234455 |
| A/chicken/Shandong/SIC39/2015 | EPI_ISL_234454 |
| A/chicken/Shandong/WF39/2016 | EPI_ISL_372501 |
| A/chicken/Shandong/WF75/2017 | EPI_ISL_327805 |
| A/chicken/ShangDong/1635/2018 | EPI_ISL_368405 |
| A/chicken/ShangDong/1646/2018 | EPI_ISL_368403 |
| A/chicken/ShangDong/16587/2018 | EPI_ISL_368402 |
| A/chicken/Shanghai/014/2014 | EPI_ISL_174447 |
| A/chicken/Shanghai/015/2014 | EPI_ISL_174449 |
| A/chicken/Shanghai/02/2015 | EPI_ISL_215582 |
| A/chicken/Shanghai/06/2015 | EPI_ISL_215583 |
| A/chicken/Shanghai/06/2018 | EPI_ISL_379840 |
| A/chicken/Shanghai/07/2018 | EPI_ISL_379841 |
| A/chicken/Shanghai/11/2018 | EPI_ISL_379842 |
| A/chicken/Shanghai/15/2015 | EPI_ISL_215584 |
| A/chicken/Shanghai/PT02/2015 | EPI_ISL_272724 |
| A/chicken/Shaoguan/zd201603/2017 | EPI_ISL_378892 |
| A/environment/Jiangsu/01/20/TCCX004/2015 | EPI_ISL_199288 |
| A/environment/Jiangsu/12/30/WZNHQ016/2014 | EPI_ISL_199338 |
| A/environment/Jiangxi/02/05/YGYXG006/2015 | EPI_ISL_199324 |
| A/environment/Jiangxi/05/06//NCJDE7/8/2015 | EPI_ISL_198951 |
| A/environment/Jiangxi/05/07//NC0048C/2015 | EPI_ISL_198979 |
| A/environment/Jiangxi/05/07/NCJD0002D/2015 | EPI_ISL_198961 |
| A/Environment/Jiangxi/10663/2014 | EPI_ISL_328593 |
| A/Environment/Jiangxi/14737/2014 | EPI_ISL_237447 |
| A/environment/Jilin/04/25/CCHL020/2015 | EPI_ISL_198839 |
| A/Environment/Nantong/nt38/2014 | EPI_ISL_170156 |
| A/Environment/Ningxia/99718/2014 | EPI_ISL_328601 |
| A/Environment/Shandong/38416/2015 | EPI_ISL_330002 |
| A/Environment/Sichuan/18597/2015 | EPI_ISL_329997 |
| A/Environment/Suzhou/sz11/2014 | EPI_ISL_170153 |
| A/environment/Wuxi/2505/2014 | EPI_ISL_277054 |
| A/environment/Wuxi/5220/2015 | EPI_ISL_277059 |
| A/Environment/Xinjiang/39018/2015 | EPI_ISL_329998 |
| A/Environment/Xuzhou/xz21/2014 | EPI_ISL_170235 |
| A/Environment/Zhenjiang/zj24/2014 | EPI_ISL_170162 |
| A/Environment/Zhenjiang/zj26/2014 | EPI_ISL_170169 |
| A/Environment/Zhenjiang/zj31/2014 | EPI_ISL_170154 |
| A/environment/Zhongshan/ZS201501/2015 | EPI_ISL_235522 |
| A/environment/Zhongshan/ZS201502/2015 | EPI_ISL_235523 |
| A/environment/Zhongshan/ZS201503/2015 | EPI_ISL_235524 |
| A/environment/Zhongshan/ZS201504/2015 | EPI_ISL_235525 |
| A/environment/Zhongshan/ZS201505/2015 | EPI_ISL_235526 |
| A/environment/Zhongshan/ZS201506/2015 | EPI_ISL_235518 |
| A/environment/Zhongshan/ZS201602/2016 | EPI_ISL_235520 |
| A/environment/Zhongshan/ZS201603/2016 | EPI_ISL_235521 |
| A/Falco/tinnunculus/Tianjin/04/2017 | EPI_ISL_397115 |
| A/goose/Guangdong/A11/2016 | EPI_ISL_305607 |
| A/goose/Wuxi/5842/2015 | EPI_ISL_277060 |
| A/Guangdong/18SF003/2018 | EPI_ISL_337277 |
| A/Guangdong/18SF064/2018 | EPI_ISL_345234 |
| A/Guangdong/MZ058/2016 | EPI_ISL_253028 |
| A/Guangxi/Xiangshan/11522/2018 | EPI_ISL_345235 |
| A/Hunan/34179/2018 | EPI_ISL_345236 |
| A/Hunan/37286/2017 | EPI_ISL_337281 |
| A/Hunan/42088/2017 | EPI_ISL_337279 |
| A/Hunan/44557/2015 | EPI_ISL_234468 |
| A/mink/Shandong/Z1/2015 | EPI_ISL_378682 |
| A/mink/Shandong/Z4/2015 | EPI_ISL_378685 |
| A/mink/Shandong/Z5/2015 | EPI_ISL_378686 |
| A/mink/Shandong/Z6/2015 | EPI_ISL_378687 |
| A/chicken/Sichuan/SIC36/2014 | EPI_ISL_234452 |
| A/chicken/Suqian/SQ1602/2016 | EPI_ISL_284666 |
| A/chicken/Taizhou/TZJF05/2015 | EPI_ISL_205131 |
| A/chicken/Wenzhou/YHQL04/2014 | EPI_ISL_205129 |
| A/chicken/Wuhan/JXQL01/2015 | EPI_ISL_205132 |
| A/chicken/Wuxi/6085/2015 | EPI_ISL_277124 |
| A/chicken/Wuxi/6088/2015 | EPI_ISL_277125 |
| A/chicken/Wuxi/6224/2015 | EPI_ISL_277126 |
| A/chicken/Wuxi/6414/2015 | EPI_ISL_277093 |
| A/chicken/Wuxi/6440/2015 | EPI_ISL_277096 |
| A/ostrich/Hebei/179/2014 | EPI_ISL_379147 |
| A/ostrich/Hebei/182/2014 | EPI_ISL_250819 |
| A/pigeon/Guangdong/04/15/SZBAXQ054/2015 | EPI_ISL_199457 |
| A/pigeon/Jilin/05/10/CCHL044/O/2015 | EPI_ISL_198850 |
| A/pigeon/Zhejiang/727044/2014 | EPI_ISL_203727 |
| A/pigeon/Zhejiang/77037/2014 | EPI_ISL_203726 |
| A/quail/Guangxi/198Q39/2015 | EPI_ISL_278206 |
| A/wild/chicken/Shanghai/C1/2014 | EPI_ISL_161829 |
| A/Zhongshan/201501/2015 | EPI_ISL_206451 |

**Table S1** The information of 33 H9N2 avian influenza virus isolated in laboratory in GISAID database.

| Name | PB2 | PB1 | PA | HA | NP | NA | M | NS |
| --- | --- | --- | --- | --- | --- | --- | --- | --- |
| A chicken Zhanjiang E531 2017 | EPI1441292 | EPI1441299 | EPI1441300 | EPI1441301 | EPI1441302 | EPI1441303 | EPI1441304 | EPI1441317 |
| A chicken Guangzhou E540 2017 | EPI1441401 | EPI1441407 | EPI1441409 | EPI1441410 | EPI1441411 | EPI1441412 | EPI1441467 | EPI1441468 |
| A chicken Shenzhen E549 2017 | EPI1441505 | EPI1441506 | EPI1441507 | EPI1441508 | EPI1441509 | EPI1441510 | EPI1441511 | EPI1441512 |
| A chicken Foshan E601 2017 | EPI1441515 | EPI1441516 | EPI1441517 | EPI1441518 | EPI1441519 | EPI1441520 | EPI1441521 | EPI144152 |
| A chicken Guangzhou E613 2017 | EPI1441571 | EPI1441572 | EPI1441573 | EPI1441574 | EPI1441575 | EPI1441576 | EPI1441577 | EPI1441578 |
| A chicken Shenzhen E699 2017 | EPI1441579 | EPI1441580 | EPI1441581 | EPI1441582 | EPI1441583 | EPI1441584 | EPI1441585 | EPI1441586 |
| A chicken Jieyang E774 2017 | EPI1441587 | EPI1441588 | EPI1441589 | EPI1441590 | EPI1441591 | EPI1441592 | EPI1441593 | EPI1441594 |
| A chicken Shanwei E776 2017 | EPI1441595 | EPI1441596 | EPI1441597 | EPI1441598 | EPI1441599 | EPI1441600 | EPI1441601 | EPI1441602 |
| A pigeon Jieyang E780 2017 | EPI1441611 | EPI1441612 | EPI1441613 | EPI1441614 | EPI1441615 | EPI1441616 | EPI1441617 | EPI1441618 |
| A chicken Guangzhou E803 2017 | EPI1441619 | EPI1441620 | EPI1441621 | EPI1441622 | EPI1441623 | EPI1441624 | EPI1441625 | EPI1441626 |
| A chicken Shenzhen E822 2017 | EPI1441627 | EPI1441628 | EPI1441629 | EPI1441635 | EPI1441717 | EPI1441758 | EPI1441783 | EPI1442005 |
| A chicken Guangzhou E923 2017 | EPI1442006 | EPI1442007 | EPI1442008 | EPI1442231 | EPI1442270 | EPI1442307 | EPI1442327 | EPI1442357 |
| A chicken Dongguan F124 2017 | EPI1443364 | EPI1443365 | EPI1443366 | EPI1443367 | EPI1443368 | EPI1443369 | EPI1443370 | EPI1443371 |
| A duck Dongguan F130 2017 | EPI1443444 | EPI1443445 | EPI1443446 | EPI1443447 | EPI1443448 | EPI1443449 | EPI1443450 | EPI1443451 |
| A chicken Foshan F1025 2018 | EPI1443436 | EPI1443437 | EPI1443438 | EPI1443439 | EPI1443440 | EPI1443441 | EPI1443442 | EPI1443443 |
| A chicken Huizhou F169 2017 | EPI1443372 | EPI1443373 | EPI1443374 | EPI1443375 | EPI1443376 | EPI1443377 | EPI1443378 | EPI1443379 |
| A chicken Guangzhou F204 2017 | EPI1443380 | EPI1443381 | EPI1443382 | EPI1443383 | EPI1443384 | EPI1443385 | EPI1443386 | EPI1443387 |
| A chicken Jiangmen F225 2018 | EPI1443388 | EPI1443389 | EPI1443390 | EPI1443391 | EPI1443392 | EPI1443393 | EPI1443394 | EPI1443395 |
| A chicken Yunfu F247 2018 | EPI1443396 | EPI1443397 | EPI1443398 | EPI1443399 | EPI1443400 | EPI1443401 | EPI1443402 | EPI1443403 |
| A chicken Shenzhen F433 2018 | EPI1443404 | EPI1443405 | EPI1443406 | EPI1443407 | EPI1443408 | EPI1443409 | EPI1443410 | EPI1443411 |
| A duck Shenzhen F460 2018 | EPI1443412 | EPI1443413 | EPI1443414 | EPI1443415 | EPI1443416 | EPI1443417 | EPI1443418 | EPI1443419 |
| A chicken Guangdong F657 2018 | EPI1443420 | EPI1443421 | EPI1443422 | EPI1443423 | EPI1443424 | EPI1443425 | EPI1443426 | EPI1443427 |
| A chicken Dongguan F674 2018 | EPI1443428 | EPI1443429 | EPI1443430 | EPI1443431 | EPI1443432 | EPI1443433 | EPI1443434 | EPI1443435 |
| A chicken Shenzhen F65 2018 | EPI1443331 | EPI1443332 | EPI1443333 | EPI1443334 | EPI1443335 | EPI1443336 | EPI1443337 | EPI1443338 |
| A chicken Shenzhen F70 2018 | EPI1443339 | EPI1443340 | EPI1443341 | EPI1443342 | EPI1443343 | EPI1443344 | EPI1443345 | EPI1443346 |
| A chicken Shenzhen F88 2018 | EPI1443356 | EPI1443357 | EPI1443358 | EPI1443359 | EPI1443360 | EPI1443361 | EPI1443362 | EPI1443363 |
| A chicken Yantai F48 2018 | EPI1846363 | EPI1846364 | EPI1846365 | EPI1846361 | EPI1846366 | EPI1846362 | EPI1846367 | EPI1846368 |
| A chicken Foshan G3 2019 | EPI1846369 | EPI1846370 | EPI1846371 | EPI1846372 | EPI1846373 | EPI1846374 | EPI1846375 | EPI1846376 |
| A chicken Guangzhou G41 2019 | EPI1846377 | EPI1846378 | EPI1846379 | EPI1846380 | EPI1846381 | EPI1846382 | EPI1846383 | EPI1846384 |
| A chicken Zhanjiang G99 2019 | EPI1846385 | EPI1846386 | EPI1846387 | EPI1846388 | EPI1846389 | EPI1846390 | EPI1846391 | EPI1846392 |
| A chicken Yunnan G130 2019 | EPI1846393 | EPI1846394 | EPI1846395 | EPI1846396 | EPI1846397 | EPI1846398 | EPI1846399 | EPI1846400 |
| A chicken Qingyuan G220 2019 | EPI1846417 | EPI1846418 | EPI1846419 | EPI1846420 | EPI1846421 | EPI1846422 | EPI1846423 | EPI1846424 |
| A duck Qingyuan G264 2019 | EPI1846425 | EPI1846426 | EPI1846427 | EPI1846428 | EPI1846429 | EPI1846430 | EPI1846431 | EPI1846432 |
