# Supplementary material for: Genetic Evolution Characteristics of Genotype G57 Virus, A Dominant Genotype of H9N2 Avian Influenza Virus
Source: Front Microbiol. 2021 Mar 3;12:633835. doi: 10.3389/fmicb.2021.633835 (PMC7965968; doi:10.3389/fmicb.2021.633835)
Supplement: Supplementary file 2 [file Table_2.docx]

**Table S2** HA gene clock model and tree prior marginal possibility of different combinations.

|  | **Molecular clock model** | **Coalescent tree prior** | **PS MLE** | **SS MLE** |
| --- | --- | --- | --- | --- |
| 2007-2012 | Strict clock | Bayesian skyline | -8480.86 | -8478.33 |
|  | Strict clock | Exponential growth | -8474.81 | -8476.60 |
|  | Strict clock | Constant size | -8488.98 | -8487.49 |
|  | Uncorrelated lognormal relaxed clock | Bayesian skyline | -8469.59 | -8472.42 |
|  | **Uncorrelated lognormal relaxed clock** | **Exponential growth** | **-8462.26** | **-8465.34** |
|  | Uncorrelated lognormal relaxed clock | Constant size | -8479.44 | -8477.79 |
| 2013-2019 | Strict clock | Bayesian skyline | -21242.96 | -21296.13 |
|  | Strict clock | Exponential growth | -21257.01 | -21263.13 |
|  | Strict clock | Constant size | -21300.15 | -21358.91 |
|  | **Uncorrelated lognormal relaxed clock** | **Bayesian skyline** | **-21126.60** | **-21177.87** |
|  | Uncorrelated lognormal relaxed clock | Exponential growth | -21131.52 | -21200.60 |
|  | Uncorrelated lognormal relaxed clock | Constant size | -21200.23 | -21243.10 |

The best-fitting tree prior and molecular clock model are indicated in bold font.

**Table S2** NA gene clock model and tree prior marginal possibility of different combinations.

|  | **Molecular clock model** | **Coalescent tree prior** | **PS MLE** | **SS MLE** |
| --- | --- | --- | --- | --- |
| 2007-2012 | Strict clock | Bayesian skyline | -7295.175 | -7301.26 |
|  | Strict clock | Exponential growth | -7296.401 | -7299.79 |
|  | Strict clock | Constant size | -7307.857 | -7307.41 |
|  | Uncorrelated lognormal relaxed clock | Bayesian skyline | -7285.914 | -7288.04 |
|  | **Uncorrelated lognormal relaxed clock** | **Exponential growth** | **-7280.658** | **-7279.32** |
|  | Uncorrelated lognormal relaxed clock | Constant size | -7293.653 | -7290.24 |
| 2013-2019 | Strict clock | Bayesian skyline | -18170.583 | -18200.91 |
|  | Strict clock | Exponential growth | -18179.019 | -18203.48 |
|  | Strict clock | Constant size | -18208.071 | -18240.82 |
|  | **Uncorrelated lognormal relaxed clock** | **Bayesian skyline** | **-18069.101** | **-18105.82** |
|  | Uncorrelated lognormal relaxed clock | Exponential growth | -18073.393 | -18129.63 |
|  | Uncorrelated lognormal relaxed clock | Constant size | -18115.413 | -18157.85 |

The best-fitting tree prior and molecular clock model are indicated in bold font.
