# Supplementary material for: Genetic Evolution Characteristics of Genotype G57 Virus, A Dominant Genotype of H9N2 Avian Influenza Virus
Source: Front Microbiol. 2021 Mar 3;12:633835. doi: 10.3389/fmicb.2021.633835 (PMC7965968; doi:10.3389/fmicb.2021.633835)
Supplement: Supplementary file 3 [file Table_3.docx]

**Table S3** Genotype diversity of H9N2 influenza viruses isolated in China from 2014 to 2019.

| Isolate | PB2 | PB1 | PA | HA | NP | NA | M | NS | Genotype |
| --- | --- | --- | --- | --- | --- | --- | --- | --- | --- |
| A chicken Guangdong 04.15SZBAXQ005 2015 | 8 | 5 | 7 | 15 | 6 | 2 | 3 | 7 | G57 |
| A chicken Guangdong 04.15SZBAXQ025 2015 | 8 | 5 | 7 | 15 | 6 | 2 | 3 | 7 | G57 |
| A chicken Guangdong 04.22DGCP100-O 2015 | 8 | 5 | 7 | 15 | 6 | 2 | 3 | 7 | G57 |
| A chicken Guangdong 04.22DGCP101-O 2015 | 8 | 5 | 7 | 15 | 6 | 1 | 3 | 7 | G118 |
| A chicken Guangdong 04.22DGCP102-O 2015 | 8 | 5 | 7 | 15 | 6 | 2 | 3 | 7 | G57 |
| A chicken Guangdong 04.23DGQTXC191-O 2015 | 8 | 5 | 7 | 15 | 6 | 1 | 3 | 7 | G118 |
| A chicken Guangdong 04.23DGQTXC191-P 2015 | 8 | 5 | 7 | 15 | 6 | 1 | 3 | 7 | G118 |
| A chicken Guangdong 04.23DGQTXC194-P 2015 | 8 | 5 | 7 | 15 | 6 | 2 | 3 | 7 | G57 |
| A Anhui Lujiang 39 2018 | 8 | 5 | 7 | 15 | 6 | 2 | 3 | 7 | G57 |
| A Anser fabalis Anhui L139 2014 | 8 | 5 | 7 | 15 | 6 | 2 | 3 | 7 | G57 |
| A Beijing 1 2016 | 8 | 5 | 7 | 15 | 6 | 2 | 3 | 7 | G57 |
| A Beijing 1 2017 | 8 | 5 | 7 | 15 | 6 | 2 | 3 | 7 | G57 |
| A chicken Anhui 03 01 FY001 O 2015 | 8 | 5 | 7 | 15 | 6 | 2 | 3 | 7 | G57 |
| A chicken Anhui AH120 2015 | 8 | 5 | 7 | 15 | 6 | 2 | 3 | 7 | G57 |
| A chicken Anhui AH326 2016 | 8 | 5 | 7 | 15 | 6 | 2 | 3 | 7 | G57 |
| A chicken Anhui AH329 2016 | 8 | 5 | 7 | 15 | 6 | 2 | 3 | 7 | G57 |
| A chicken Anhui AH450 2017 | 8 | 5 | 7 | 15 | 6 | 2 | 3 | 7 | G57 |
| A chicken Anhui AH480 2017 | 8 | 5 | 7 | 15 | 6 | 2 | 3 | 7 | G57 |
| A chicken Anhui LH66 2017 | 8 | 5 | 7 | 15 | 6 | 2 | 3 | 7 | G57 |
| A chicken Anhui LH99 2017 | 8 | 5 | 7 | 15 | 6 | 2 | 3 | 7 | G57 |
| A chicken Anhui WB 2014 | 8 | 5 | 7 | 15 | 6 | 2 | 3 | 7 | G57 |
| A chicken China ShangDong 2018 | 8 | 5 | 7 | 15 | 6 | 2 | 3 | 7 | G57 |
| A chicken Daye DY0602 2017 | 8 | 5 | 7 | 15 | 6 | 2 | 3 | 7 | G57 |
| A chicken Dongguan 1674 2014 | 8 | 5 | 7 | 15 | 6 | 2 | 3 | 7 | G57 |
| A chicken Fujian S1XA35 2017 | 8 | 5 | 7 | 15 | 6 | 2 | 3 | 7 | G57 |
| A chicken Fujian SD037 2017 | 8 | 5 | 7 | 15 | 6 | 2 | 3 | 7 | G57 |
| A chicken Fujian SD056 2017 | 8 | 5 | 7 | 15 | 6 | 2 | 3 | 7 | G57 |
| A chicken Fujian SD070 2017 | 8 | 5 | 7 | 15 | 6 | 2 | 3 | 7 | G57 |
| A chicken Fujian SIC16 2014 | 8 | 5 | 7 | 15 | 6 | 2 | 3 | 7 | G57 |
| A chicken Ganzhou GZ126 2016 | 8 | 5 | 7 | 15 | 6 | 2 | 3 | 7 | G57 |
| A chicken Ganzhou GZ140 2016 | 8 | 5 | 7 | 15 | 6 | 2 | 3 | 7 | G57 |
| A chicken Ganzhou GZ86 2016 | 8 | 5 | 7 | 15 | 6 | 2 | 3 | 7 | G57 |
| A chicken Guangdong 835 2016 | 8 | 5 | 7 | 15 | 6 | 2 | 3 | 7 | G57 |
| A chicken Guangdong GD1601 2016 | 8 | 5 | 7 | 15 | 6 | 2 | 3 | 7 | G57 |
| A chicken Guangdong SIC17 2014 | 8 | 5 | 7 | 15 | 6 | 2 | 3 | 7 | G57 |
| A chicken Guangdong SIC18 2014 | 8 | 5 | 7 | 15 | 6 | 2 | 3 | 7 | G57 |
| A chicken Guangdong SIC23 2014 | 8 | 5 | 7 | 15 | 6 | 2 | 3 | 7 | G57 |
| A chicken Guangdong SIC28 2014 | 8 | 5 | 7 | 15 | 6 | 2 | 3 | 7 | G57 |
| A chicken Guangdong SIC29 2014 | 8 | 5 | 7 | 15 | 6 | 2 | 3 | 7 | G57 |
| A chicken Wuxi 6468 2015 | 8 | 5 | 7 | 15 | 6 | 2 | 3 | 7 | G57 |
| A chicken Wuxi 6657 2015 | 8 | 5 | 7 | 15 | 6 | 2 | 3 | 7 | G57 |
| A chicken Wuxi 6688 2015 | 8 | 5 | 7 | 15 | 6 | 2 | 3 | 7 | G57 |
| A chicken Wuxi 7109 2015 | 8 | 5 | 7 | 15 | 6 | 2 | 3 | 7 | G57 |
| A chicken Wuxi 8501 2016 | 8 | 5 | 7 | 15 | 6 | 2 | 3 | 7 | G57 |
| A chicken Wuxi SC4315 2015 | 8 | 5 | 7 | 15 | 6 | 2 | 3 | 7 | G57 |
| A chicken Wuxi7723 2016 | 8 | 5 | 7 | 15 | 6 | 2 | 3 | 7 | G57 |
| A chicken Xiamen 10 2015 | 8 | 5 | 7 | 15 | 6 | 2 | 3 | 7 | G57 |
| A chicken Xuancheng 01 2018 | 8 | 5 | 7 | 15 | 6 | 2 | 3 | 7 | G57 |
| A chicken Xuzhou XZ270 2016 | 8 | 5 | 7 | 15 | 6 | 2 | 3 | 7 | G57 |
| A chicken Yunan 07 13 DQDBS051 2015 | 8 | 5 | 7 | 15 | 6 | 2 | 3 | 7 | G57 |
| A chicken Yunnan 03 15 DQWGH005 Z O 2015 | 8 | 5 | 7 | 15 | 6 | 2 | 3 | 7 | G57 |
| A chicken Yunnan 03 15 DQXYL0029 O 2015 | 8 | 5 | 7 | 15 | 6 | 2 | 3 | 7 | G57 |
| A chicken Yunnan 03 16 DQJT0070 O 2015 | 8 | 5 | 7 | 15 | 6 | 2 | 3 | 7 | G57 |
| A chicken Yunnan 03 16 DQJT0071 O 2015 | 8 | 5 | 7 | 15 | 6 | 2 | 3 | 7 | G57 |
| A chicken Yunnan 03 16 DQJT062 O 2015 | 8 | 5 | 7 | 15 | 6 | 2 | 3 | 7 | G57 |
| A chicken Yunnan 03 16 DQXYL0031 O 2015 | 8 | 5 | 7 | 15 | 6 | 2 | 3 | 7 | G57 |
| A chicken Zhejiang 221 2016 | 8 | 5 | 7 | 15 | 6 | 2 | 3 | 7 | G57 |
| A chicken Zhejiang 3C34 2014 | 8 | 5 | 7 | 15 | 6 | 2 | 3 | 7 | G57 |
| A chicken Zhejiang 727063 2014 | 8 | 5 | 7 | 15 | 6 | 2 | 3 | 7 | G57 |
| A chicken Zhejiang 727192 2014 | 8 | 5 | 7 | 15 | 6 | 2 | 3 | 7 | G57 |
| A chicken Zhejiang 727198 2014 | 8 | 5 | 7 | 15 | 6 | 2 | 3 | 7 | G57 |
| A chicken Zhejiang 77082 2014 | 8 | 5 | 7 | 15 | 6 | 2 | 3 | 7 | G57 |
| A chicken Zhejiang 925060 2014 | 8 | 5 | 7 | 15 | 6 | 2 | 3 | 7 | G57 |
| A chicken Zhejiang 925117 2014 | 8 | 5 | 7 | 15 | 6 | 2 | 3 | 7 | G57 |
| A chicken Zhejiang 925122 2014 | 8 | 5 | 7 | 15 | 6 | 2 | 3 | 7 | G57 |
| A chicken Zhejiang 925159 2014 | 8 | 5 | 7 | 15 | 6 | 2 | 3 | 7 | G57 |
| A chicken Zhejiang SIC30 2014 | 8 | 5 | 7 | 15 | 6 | 2 | 3 | 7 | G57 |
| A chicken Zhejiang SIC32 2014 | 8 | 5 | 7 | 15 | 6 | 2 | 3 | 7 | G57 |
| A chicken Zhejiang SIC40 2015 | 8 | 5 | 7 | 15 | 6 | 2 | 3 | 7 | G57 |
| A chicken Zhejiang TL27 2014 | 8 | 5 | 7 | 15 | 6 | 2 | 3 | 7 | G57 |
| A dove Guangxi 96B8 2014 | 8 | 5 | 7 | 4 | 6 | 3 | 3 | 7 | G121 |
| A duck Anhui AQ14 2014 | 8 | 5 | 7 | 15 | 6 | 2 | 3 | 7 | G57 |
| A duck Ganzhou GZ188 2016 | 8 | 5 | 7 | 15 | 6 | 2 | 3 | 7 | G57 |
| A duck Guangdong 03 26 DGCP101 O 2015 | 8 | 5 | 7 | 15 | 6 | 1 | 3 | 7 | G118 |
| A duck Guangdong 03 26 DGCP115 O 2015 | 8 | 5 | 7 | 15 | 6 | 2 | 3 | 7 | G57 |
| A duck Guangdong 222 2015 | 8 | 5 | 7 | 12 | 6 | 2 | 3 | 7 | G68 |
| A duck Guangdong A9 2016 | 8 | 5 | 7 | 12 | 6 | 2 | 3 | 7 | G68 |
| A duck Hubei 03 06 WHWTZ0140 P 2015 | 8 | 5 | 7 | 15 | 6 | 2 | 3 | 7 | G57 |
| A chicken Guangdong SIC31 2014 | 8 | 5 | 7 | 15 | 6 | 2 | 3 | 7 | G57 |
| A chicken Guangdong SIC37 2015 | 8 | 5 | 7 | 15 | 6 | 2 | 3 | 7 | G57 |
| A chicken Guangdong SIC38 2015 | 8 | 5 | 7 | 15 | 6 | 2 | 3 | 7 | G57 |
| A chicken Guangdong SIC41 2015 | 8 | 5 | 7 | 15 | 6 | 2 | 3 | 7 | G57 |
| A chicken Guangxi C1228 2015 | 8 | 5 | 7 | 15 | 6 | 2 | 3 | 7 | G57 |
| A chicken Guangxi C227 2015 | 8 | 5 | 7 | 15 | 6 | 2 | 3 | 7 | G57 |
| A chicken Guangxi SIC19 2014 | 8 | 5 | 7 | 15 | 6 | 1 | 3 | 7 | G118 |
| A chicken Guangxi SIC20 2014 | 8 | 5 | 7 | 15 | 6 | 2 | 3 | 7 | G57 |
| A chicken Guangxi SIC22 2014 | 8 | 5 | 7 | 15 | 6 | 2 | 3 | 7 | G57 |
| A chicken Hainan SIC33 2014 | 4 | 5 | 7 | 15 | 6 | 2 | 3 | 7 | G119 |
| A chicken Henan 815 2016 | 8 | 5 | 7 | 15 | 6 | 2 | 3 | 7 | G57 |
| A chicken Huaian HA9 2016 | 8 | 5 | 7 | 15 | 6 | 2 | 3 | 7 | G57 |
| A chicken Hubei 01 2015 | 8 | 5 | 7 | 15 | 6 | 2 | 3 | 7 | G57 |
| A chicken Hubei 03 06 WHWTZ0068 P 2015 | 8 | 5 | 7 | 15 | 6 | 2 | 3 | 7 | G57 |
| A chicken Hubei 03 06 WHWTZ0088 P 2015 | 8 | 5 | 7 | 15 | 6 | 2 | 3 | 7 | G57 |
| A chicken Hubei 03 06 WHWTZ0048 P 2015 | 8 | 5 | 7 | 15 | 6 | 2 | 3 | 7 | G57 |
| A chicken Hubei 2014 | 8 | 5 | 7 | 15 | 6 | 2 | 3 | 7 | G57 |
| A chicken Hubei ZYSJF15 2016 | 8 | 5 | 7 | 15 | 6 | 2 | 3 | 7 | G57 |
| A chicken Hunan 04 14 YYGK501 O 2015 | 8 | 5 | 7 | 15 | 6 | 2 | 3 | 7 | G57 |
| A chicken Hunan 04 14 YYGK506 O 2015 | 8 | 5 | 7 | 15 | 6 | 2 | 3 | 7 | G57 |
| A chicken Hunan 04 14 YYGK507 O 2015 | 8 | 5 | 7 | 15 | 6 | 2 | 3 | 7 | G57 |
| A chicken Hunan 04 14 YYGK563 P 2015 | 8 | 5 | 7 | 15 | 6 | 2 | 3 | 7 | G57 |
| A chicken Hunan 04 22 LDDX046 O 2015 | 8 | 5 | 7 | 15 | 6 | 2 | 3 | 7 | G57 |
| A chicken Hunan 04 22 LDDX069 O 2015 | 8 | 5 | 7 | 15 | 6 | 2 | 3 | 7 | G57 |
| A chicken Hunan 12 17 YYFQH0015 O 2014 | 8 | 5 | 7 | 15 | 6 | 2 | 3 | 7 | G57 |
| A chicken Hunan YYFQH689 O 2015 | 8 | 5 | 7 | 15 | 6 | 2 | 3 | 7 | G57 |
| A chicken Jiangsu 02 06 NJLC042 O 2015 | 8 | 5 | 7 | 15 | 6 | 2 | 3 | 7 | G57 |
| A chicken Jiangsu 02 06 NJLC068 O 2015 | 8 | 5 | 7 | 15 | 6 | 2 | 3 | 7 | G57 |
| A chicken Jiangsu 03 06 WXBT054 O 2015 | 8 | 5 | 7 | 15 | 6 | 2 | 3 | 7 | G57 |
| A chicken Jiangsu 12 30 WZNHQ031 P 2014 | 8 | 5 | 7 | 15 | 6 | 2 | 3 | 7 | G57 |
| A chicken Jiangsu JS4539 2014 | 8 | 5 | 7 | 15 | 6 | 2 | 3 | 7 | G57 |
| A chicken Jiangsu JT138 2016 | 8 | 5 | 7 | 15 | 6 | 2 | 3 | 7 | G57 |
| A chicken Jiangsu JT141 2016 | 8 | 5 | 7 | 15 | 6 | 2 | 3 | 7 | G57 |
| A chicken Jiangsu JT154 2016 | 8 | 5 | 7 | 15 | 6 | 2 | 3 | 7 | G57 |
| A chicken Jiangsu LY2 2017 | 8 | 5 | 7 | 15 | 6 | 2 | 3 | 7 | G57 |
| A chicken Jiangsu SIC42 2015 | 8 | 5 | 7 | 15 | 6 | 2 | 3 | 7 | G57 |
| A chicken Jiangsu TM306 2017 | 8 | 5 | 7 | 15 | 6 | 2 | 3 | 7 | G57 |
| A chicken Jiangsu TM314 2017 | 8 | 5 | 7 | 15 | 6 | 2 | 3 | 7 | G57 |
| A chicken Jiangsu TM315 2017 | 8 | 5 | 7 | 15 | 6 | 2 | 3 | 7 | G57 |
| A chicken Jiangsu TM71 2014 | 8 | 5 | 7 | 15 | 6 | 2 | 3 | 7 | G57 |
| A chicken Jiangsu WJ179 2015 | 8 | 5 | 7 | 15 | 6 | 2 | 3 | 7 | G57 |
| A chicken Jiangxi 04 01 NCDTZ0261 P 2015 | 8 | 5 | 7 | 15 | 6 | 2 | 3 | 7 | G57 |
| A chicken Jiangxi 04 01 NCDZT0055 O 2015 | 8 | 5 | 7 | 15 | 6 | 2 | 3 | 7 | G57 |
| A chicken Jiangxi 04 01 NCDZT0103 O 2015 | 8 | 5 | 7 | 15 | 6 | 2 | 3 | 7 | G57 |
| A duck Hunan 01 16 YYGK225 P 2014 | 8 | 5 | 7 | 15 | 6 | 2 | 3 | 7 | G57 |
| A duck Hunan 02 26 YYGK250 P 2014 | 8 | 5 | 7 | 15 | 6 | 2 | 3 | 7 | G57 |
| A duck Hunan 04 14 YYXS0888 2 P 2015 | 8 | 5 | 7 | 15 | 6 | 2 | 3 | 7 | G57 |
| A duck Hunan 04 14 YYGK443 P 2015 | 8 | 5 | 7 | 15 | 6 | 2 | 3 | 7 | G57 |
| A duck Hunan 04 14 YYGK901 O 2015 | 8 | 5 | 7 | 15 | 6 | 2 | 3 | 7 | G57 |
| A duck Hunan 4 14 YYGK0453 2 O 2015 | 8 | 5 | 7 | 15 | 6 | 2 | 3 | 7 | G57 |
| A duck Jiangxi 05 07 NCJD0030A O 2015 | 8 | 5 | 7 | 15 | 6 | 2 | 3 | 7 | G57 |
| A duck ShanDong JN1 2015 | 6 | 5 | 7 | 15 | 6 | 2 | 2 | 0 | G123 |
| A duck Wenzhou YHQL64 2014 | 8 | 5 | 7 | 15 | 6 | 2 | 3 | 7 | G57 |
| A duck Wuhan WHYF05 2014 | 8 | 2 | 5 | 2 | 1 | 0 | 1 | 0 | G122 |
| A duck Wuhan WHYF14 2014 | 8 | 5 | 7 | 15 | 6 | 2 | 3 | 7 | G57 |
| A duck Wuxi 5083 2015 | 8 | 5 | 7 | 15 | 6 | 2 | 3 | 7 | G57 |
| A duck Wuxi 6663 2015 | 8 | 5 | 7 | 15 | 6 | 2 | 3 | 7 | G57 |
| A duck Yunnan 03 16 DQXYL007 Z O 2015 | 8 | 5 | 7 | 15 | 6 | 2 | 3 | 7 | G57 |
| A Environment Anhui 09183 2014 | 8 | 5 | 7 | 15 | 6 | 2 | 3 | 7 | G57 |
| A Environment Anhui 33231 2015 | 8 | 5 | 7 | 15 | 6 | 2 | 3 | 7 | G57 |
| A environment Beijing 1 2016 | 8 | 5 | 7 | 15 | 6 | 2 | 3 | 7 | G57 |
| A environment Beijing 2 2016 | 8 | 5 | 7 | 15 | 6 | 2 | 3 | 7 | G57 |
| A environment Beijing 5 2016 | 8 | 5 | 7 | 15 | 6 | 2 | 3 | 7 | G57 |
| A Environment Changzhou cz96 2014 | 8 | 5 | 7 | 15 | 6 | 2 | 3 | 7 | G57 |
| A Environment Chongqing 74343 2014 | 8 | 5 | 7 | 15 | 6 | 2 | 3 | 7 | G57 |
| A Environment Fujian 07241 2014 | 8 | 5 | 7 | 15 | 6 | 2 | 3 | 7 | G57 |
| A Environment Fujian 85145 2014 | 8 | 5 | 7 | 15 | 6 | 2 | 3 | 7 | G57 |
| A Environment Gansu 99800 2014 | 8 | 5 | 7 | 15 | 6 | 2 | 3 | 7 | G57 |
| A environment Guangdong 03 27 DGQTSJ042 2015 | 8 | 5 | 7 | 15 | 6 | 2 | 3 | 7 | G57 |
| A environment Guangdong 04 22 DGCPLB005 2015 | 8 | 5 | 7 | 15 | 6 | 2 | 3 | 7 | G57 |
| A Environment Guangdong 21115 2015 | 8 | 5 | 7 | 4 | 6 | 3 | 3 | 7 | G121 |
| A Environment Guangdong 34438 2015 | 8 | 5 | 7 | 15 | 6 | 2 | 3 | 7 | G57 |
| A Environment Guangdong 38697 2015 | 8 | 5 | 7 | 15 | 6 | 1 | 3 | 7 | G118 |
| A Environment Guangdong 72907 2014 | 8 | 5 | 7 | 15 | 6 | 2 | 3 | 7 | G57 |
| A Environment Guangdong 98640 2014 | 8 | 5 | 7 | 15 | 6 | 2 | 3 | 7 | G57 |
| A Environment Guangxi 32197 2014 | 8 | 5 | 7 | 15 | 6 | 2 | 3 | 7 | G57 |
| A Environment Guangxi 47379 2014 | 8 | 5 | 7 | 15 | 6 | 2 | 3 | 7 | G57 |
| A Environment Henan 98615 2014 | 8 | 5 | 7 | 15 | 6 | 2 | 3 | 7 | G57 |
| A Environment Hunan 00824 2014 | 8 | 5 | 7 | 15 | 6 | 2 | 3 | 7 | G57 |
| A environment Hunan 04 14 YYGK388 2015 | 8 | 5 | 7 | 15 | 6 | 2 | 3 | 7 | G57 |
| A environment Hunan 04 14 YYGK400 2015 | 8 | 5 | 7 | 15 | 6 | 2 | 3 | 7 | G57 |
| A environment Hunan 26018 2014 | 8 | 5 | 7 | 15 | 6 | 2 | 3 | 7 | G57 |
| A environment Hunan 28176 2014 | 8 | 5 | 7 | 15 | 6 | 2 | 3 | 7 | G57 |
| A environment Hunan 28184 2014 | 8 | 5 | 7 | 15 | 6 | 2 | 3 | 7 | G57 |
| A Environment Hunan 32371 2015 | 8 | 5 | 7 | 15 | 6 | 2 | 3 | 7 | G57 |
| A Environment Hunan 32438 2015 | 8 | 5 | 7 | 15 | 6 | 2 | 3 | 7 | G57 |
| A Environment Hunan 39658 2015 | 8 | 5 | 7 | 15 | 6 | 2 | 3 | 7 | G57 |
| A Environment Hunan 39729 2015 | 8 | 5 | 7 | 15 | 6 | 2 | 3 | 7 | G57 |
| A chicken Jiangxi 04 01 NCJD0106 O 2015 | 8 | 5 | 7 | 15 | 6 | 2 | 3 | 7 | G57 |
| A chicken Jiangxi 05 06 NCDZT0077B P 2015 | 8 | 5 | 7 | 15 | 6 | 2 | 3 | 7 | G57 |
| A chicken Jiangxi 1202 2014 | 8 | 5 | 7 | 15 | 6 | 2 | 3 | 7 | G57 |
| A chicken Jilin 04 04 SY001 O 2015 | 8 | 5 | 7 | 15 | 6 | 2 | 3 | 7 | G57 |
| A chicken Jilin 04 05 CCCJ006 2015 | 8 | 5 | 7 | 15 | 6 | 2 | 3 | 7 | G57 |
| A Chicken Jilin 13200 2014 | 8 | 5 | 7 | 15 | 6 | 2 | 3 | 7 | G57 |
| A Chicken Jilin 13204 2014 | 8 | 5 | 7 | 15 | 6 | 2 | 3 | 7 | G57 |
| A chicken Jilin SD001 2014 | 8 | 5 | 7 | 15 | 6 | 2 | 1 | 7 | G120 |
| A chicken Jingmen JM0305 2017 | 8 | 5 | 7 | 15 | 6 | 2 | 3 | 7 | G57 |
| A chicken Qingdao 003 2014 | 8 | 5 | 7 | 15 | 6 | 2 | 3 | 7 | G57 |
| A chicken Qingdao 008 2014 | 8 | 5 | 7 | 15 | 6 | 2 | 3 | 7 | G57 |
| A chicken Qingdao 009 2014 | 8 | 5 | 7 | 15 | 6 | 2 | 3 | 7 | G57 |
| A chicken Qingdao 013 2014 | 8 | 5 | 7 | 15 | 6 | 2 | 3 | 7 | G57 |
| A chicken Qingdao 015 2014 | 8 | 5 | 7 | 15 | 6 | 2 | 3 | 7 | G57 |
| A chicken Qingdao 017 2014 | 8 | 5 | 7 | 15 | 6 | 2 | 3 | 7 | G57 |
| A chicken Qingdao 020 2014 | 8 | 5 | 7 | 15 | 6 | 2 | 3 | 7 | G57 |
| A chicken Qingyuan zd201602 2016 | 8 | 5 | 7 | 15 | 6 | 2 | 3 | 7 | G57 |
| A chicken Shandong 1167 2015 | 8 | 5 | 7 | 15 | 6 | 2 | 3 | 7 | G57 |
| A chicken ShanDong 210WZ 2017 | 8 | 5 | 7 | 15 | 6 | 2 | 3 | 7 | G57 |
| A chicken ShanDong 306SZ 2017 | 8 | 5 | 7 | 15 | 6 | 2 | 3 | 7 | G57 |
| A chicken ShanDong 321ZL 2017 | 8 | 5 | 7 | 15 | 6 | 2 | 3 | 7 | G57 |
| A chicken ShanDong 413ZDM 2017 | 8 | 5 | 7 | 15 | 6 | 2 | 3 | 7 | G57 |
| A chicken Shandong LY1 2017 | 6 | 5 | 7 | 5 | 6 | 2 | 2 | 0 | G124 |
| A chicken Shandong SIC24 2014 | 8 | 5 | 7 | 15 | 6 | 2 | 3 | 7 | G57 |
| A chicken Shandong SIC25 2014 | 8 | 5 | 7 | 15 | 6 | 2 | 3 | 7 | G57 |
| A chicken Shandong SIC26 2014 | 8 | 5 | 7 | 15 | 6 | 2 | 3 | 7 | G57 |
| A chicken Shandong SIC34 2014 | 8 | 5 | 7 | 15 | 6 | 2 | 3 | 7 | G57 |
| A chicken Shandong SIC35 2014 | 8 | 5 | 7 | 15 | 6 | 2 | 3 | 7 | G57 |
| A chicken Shandong SIC39 2015 | 8 | 5 | 7 | 15 | 6 | 2 | 3 | 7 | G57 |
| A chicken Shandong WF39 2016 | 8 | 5 | 7 | 15 | 6 | 2 | 3 | 7 | G57 |
| A chicken Shandong WF75 2017 | 8 | 5 | 7 | 15 | 6 | 2 | 3 | 7 | G57 |
| A chicken ShangDong 1635 2018 | 8 | 5 | 7 | 15 | 6 | 2 | 3 | 7 | G57 |
| A chicken ShangDong 1646 2018 | 8 | 5 | 7 | 15 | 6 | 2 | 3 | 7 | G57 |
| A chicken ShangDong 16587 2018 | 8 | 5 | 7 | 15 | 6 | 2 | 3 | 7 | G57 |
| A chicken Shanghai 014 2014 | 8 | 5 | 7 | 15 | 6 | 2 | 3 | 7 | G57 |
| A chicken Shanghai 015 2014 | 8 | 5 | 7 | 15 | 6 | 2 | 3 | 7 | G57 |
| A chicken Shanghai 02 2015 | 8 | 5 | 7 | 15 | 6 | 2 | 3 | 7 | G57 |
| A chicken Shanghai 06 2015 | 8 | 5 | 7 | 15 | 6 | 2 | 3 | 7 | G57 |
| A chicken Shanghai 06 2018 | 8 | 5 | 7 | 15 | 6 | 2 | 3 | 7 | G57 |
| A chicken Shanghai 07 2018 | 8 | 5 | 7 | 15 | 6 | 2 | 3 | 7 | G57 |
| A chicken Shanghai 11 2018 | 8 | 5 | 7 | 15 | 6 | 2 | 3 | 7 | G57 |
| A chicken Shanghai 15 2015 | 8 | 5 | 7 | 15 | 6 | 2 | 3 | 7 | G57 |
| A chicken Shanghai PT02 2015 | 8 | 5 | 7 | 15 | 6 | 2 | 3 | 7 | G57 |
| A chicken Shaoguan zd201603 2017 | 8 | 5 | 7 | 15 | 6 | 2 | 0 | 7 | G125 |
| A environment Jiangsu 01 20 TCCX004 2015 | 8 | 5 | 7 | 15 | 6 | 2 | 3 | 7 | G57 |
| A environment Jiangsu 12 30 WZNHQ016 2014 | 8 | 5 | 7 | 15 | 6 | 2 | 3 | 7 | G57 |
| A environment Jiangxi 02 05 YGYXG006 2015 | 8 | 5 | 7 | 15 | 6 | 2 | 3 | 7 | G57 |
| A environment Jiangxi 05 06 NCJDE7 8 2015 | 8 | 5 | 7 | 15 | 6 | 2 | 3 | 7 | G57 |
| A environment Jiangxi 05 07 NC0048C 2015 | 8 | 5 | 7 | 15 | 6 | 2 | 3 | 7 | G57 |
| A environment Jiangxi 05 07 NCJD0002D 2015 | 8 | 5 | 7 | 15 | 6 | 2 | 3 | 7 | G57 |
| A Environment Jiangxi 10663 2014 | 8 | 5 | 7 | 15 | 6 | 2 | 3 | 7 | G57 |
| A Environment Jiangxi 14737 2014 | 8 | 5 | 7 | 15 | 6 | 2 | 3 | 7 | G57 |
| A environment Jilin 04 25 CCHL020 2015 | 8 | 5 | 7 | 15 | 6 | 2 | 3 | 7 | G57 |
| A Environment Nantong nt38 2014 | 8 | 5 | 7 | 15 | 6 | 2 | 3 | 7 | G57 |
| A Environment Ningxia 99718 2014 | 8 | 5 | 7 | 15 | 6 | 2 | 3 | 7 | G57 |
| A Environment Shandong 38416 2015 | 8 | 5 | 7 | 15 | 6 | 2 | 3 | 7 | G57 |
| A Environment Sichuan 18597 2015 | 8 | 5 | 7 | 15 | 6 | 2 | 3 | 7 | G57 |
| A Environment Suzhou sz11 2014 | 8 | 5 | 7 | 15 | 6 | 2 | 3 | 7 | G57 |
| A environment Wuxi 2505 2014 | 8 | 5 | 7 | 15 | 6 | 2 | 3 | 7 | G57 |
| A environment Wuxi 5220 2015 | 8 | 5 | 7 | 15 | 6 | 2 | 3 | 7 | G57 |
| A Environment Xinjiang 39018 2015 | 8 | 5 | 7 | 15 | 6 | 2 | 3 | 7 | G57 |
| A Environment Xuzhou xz21 2014 | 8 | 5 | 7 | 15 | 6 | 2 | 3 | 7 | G57 |
| A Environment Zhenjiang zj24 2014 | 8 | 5 | 7 | 15 | 6 | 2 | 3 | 7 | G57 |
| A Environment Zhenjiang zj26 2014 | 8 | 5 | 7 | 15 | 6 | 2 | 3 | 7 | G57 |
| A Environment Zhenjiang zj31 2014 | 8 | 5 | 7 | 15 | 6 | 2 | 3 | 7 | G57 |
| A environment Zhongshan ZS201501 2015 | 8 | 5 | 7 | 15 | 6 | 2 | 3 | 7 | G57 |
| A environment Zhongshan ZS201502 2015 | 8 | 5 | 7 | 15 | 6 | 2 | 3 | 7 | G57 |
| A environment Zhongshan ZS201503 2015 | 8 | 5 | 7 | 15 | 6 | 2 | 3 | 7 | G57 |
| A environment Zhongshan ZS201504 2015 | 8 | 5 | 7 | 15 | 6 | 2 | 3 | 7 | G57 |
| A environment Zhongshan ZS201505 2015 | 8 | 5 | 7 | 15 | 6 | 1 | 3 | 7 | G118 |
| A environment Zhongshan ZS201506 2015 | 8 | 5 | 7 | 15 | 6 | 2 | 3 | 7 | G57 |
| A environment Zhongshan ZS201602 2016 | 8 | 5 | 7 | 15 | 6 | 2 | 3 | 7 | G57 |
| A environment Zhongshan ZS201603 2016 | 8 | 5 | 7 | 15 | 6 | 2 | 3 | 7 | G57 |
| A Falco tinnunculus Tianjin 04 2017 | 8 | 5 | 7 | 15 | 6 | 2 | 3 | 7 | G57 |
| A goose Guangdong A11 2016 | 8 | 5 | 7 | 15 | 6 | 2 | 3 | 7 | G57 |
| A goose Wuxi 5842 2015 | 8 | 5 | 7 | 15 | 6 | 2 | 3 | 7 | G57 |
| A Guangdong 18SF003 2018 | 8 | 5 | 7 | 15 | 6 | 2 | 3 | 7 | G57 |
| A Guangdong 18SF064 2018 | 8 | 5 | 7 | 15 | 6 | 2 | 3 | 7 | G57 |
| A Guangdong MZ058 2016 | 8 | 5 | 7 | 15 | 6 | 2 | 3 | 7 | G57 |
| A Guangxi Xiangshan 11522 2018 | 8 | 5 | 7 | 15 | 6 | 2 | 3 | 7 | G57 |
| A Hunan 34179 2018 | 8 | 5 | 7 | 15 | 6 | 2 | 3 | 7 | G57 |
| A Hunan 37286 2017 | 8 | 5 | 7 | 15 | 6 | 2 | 3 | 7 | G57 |
| A Hunan 42088 2017 | 8 | 5 | 7 | 15 | 6 | 2 | 3 | 7 | G57 |
| A Hunan 44557 2015 | 8 | 5 | 7 | 15 | 6 | 2 | 3 | 7 | G57 |
| A mink Shandong Z1 2015 | 8 | 5 | 7 | 15 | 6 | 2 | 3 | 7 | G57 |
| A mink Shandong Z4 2015 | 8 | 5 | 7 | 15 | 6 | 2 | 3 | 7 | G57 |
| A mink Shandong Z5 2015 | 8 | 5 | 7 | 15 | 6 | 2 | 3 | 7 | G57 |
| A mink Shandong Z6 2015 | 8 | 5 | 7 | 15 | 6 | 2 | 3 | 7 | G57 |
| A chicken Sichuan SIC36 2014 | 8 | 5 | 7 | 15 | 6 | 2 | 3 | 7 | G57 |
| A chicken Suqian SQ1602 2016 | 8 | 5 | 7 | 15 | 6 | 2 | 3 | 7 | G57 |
| A chicken Taizhou TZJF05 2015 | 8 | 5 | 7 | 15 | 6 | 2 | 3 | 7 | G57 |
| A chicken Wenzhou YHQL04 2014 | 8 | 5 | 7 | 15 | 6 | 2 | 3 | 7 | G57 |
| A chicken Wuhan JXQL01 2015 | 8 | 5 | 7 | 15 | 6 | 2 | 3 | 7 | G57 |
| A chicken Wuxi 6085 2015 | 8 | 5 | 7 | 15 | 6 | 2 | 3 | 7 | G57 |
| A chicken Wuxi 6088 2015 | 8 | 5 | 7 | 15 | 6 | 2 | 3 | 7 | G57 |
| A chicken Wuxi 6224 2015 | 8 | 5 | 7 | 15 | 6 | 2 | 3 | 7 | G57 |
| A chicken Wuxi 6414 2015 | 8 | 5 | 7 | 15 | 6 | 2 | 3 | 7 | G57 |
| A chicken Wuxi 6440 2015 | 8 | 5 | 7 | 15 | 6 | 2 | 3 | 7 | G57 |
| A ostrich Hebei 179 2014 | 8 | 5 | 7 | 15 | 6 | 2 | 3 | 7 | G57 |
| A ostrich Hebei 182 2014 | 8 | 5 | 7 | 15 | 6 | 2 | 3 | 7 | G57 |
| A pigeon Guangdong 04 15 SZBAXQ054 2015 | 8 | 5 | 7 | 15 | 6 | 2 | 3 | 7 | G57 |
| A pigeon Jilin 05 10 CCHL044 O 2015 | 8 | 5 | 7 | 15 | 6 | 2 | 3 | 7 | G57 |
| A pigeon Zhejiang 727044 2014 | 8 | 5 | 7 | 15 | 6 | 2 | 3 | 7 | G57 |
| A pigeon Zhejiang 77037 2014 | 8 | 5 | 7 | 15 | 6 | 2 | 3 | 7 | G57 |
| A quail Guangxi 198Q39 2015 | 8 | 5 | 7 | 15 | 6 | 2 | 3 | 7 | G57 |
| A wild chicken Shanghai C1 2014 | 8 | 5 | 7 | 15 | 6 | 2 | 3 | 7 | G57 |
| A Zhongshan 201501 2015 | 8 | 5 | 7 | 15 | 6 | 2 | 3 | 7 | G57 |
| A chicken Zhanjiang E531 2017 | 8 | 5 | 7 | 15 | 6 | 2 | 3 | 7 | G57 |
| A chicken Guangdong E540 2017 | 8 | 5 | 7 | 15 | 6 | 2 | 3 | 7 | G57 |
| A chicken Shenzhen E549 2017 | 8 | 5 | 7 | 15 | 6 | 2 | 3 | 7 | G57 |
| A chicken Foshan E601 2017 | 8 | 5 | 7 | 15 | 6 | 2 | 3 | 7 | G57 |
| A chicken Guangzhou E613 2017 | 8 | 5 | 7 | 15 | 6 | 2 | 3 | 7 | G57 |
| A chicken Shenzhen E699 2017 | 8 | 5 | 7 | 15 | 6 | 2 | 3 | 7 | G57 |
| A chicken Jieyang E774 2017 | 8 | 5 | 7 | 15 | 6 | 2 | 3 | 7 | G57 |
| A chicken Shanwei E776 2017 | 8 | 5 | 7 | 15 | 6 | 2 | 3 | 7 | G57 |
| A pigeon Jieyang E780 2017 | 8 | 5 | 7 | 15 | 6 | 2 | 3 | 7 | G57 |
| A chicken Guangzhou E803 2017 | 8 | 5 | 7 | 15 | 6 | 2 | 3 | 7 | G57 |
| A chicken Shenzhen E822 2017 | 8 | 5 | 7 | 15 | 6 | 2 | 3 | 7 | G57 |
| A chicken Guangzhou E923 2017 | 8 | 5 | 7 | 15 | 6 | 2 | 3 | 7 | G57 |
| A chicken Dongguan F124 2017 | 8 | 5 | 7 | 15 | 6 | 2 | 3 | 7 | G57 |
| A duck Dongguan F130 2017 | 8 | 5 | 7 | 15 | 6 | 2 | 3 | 7 | G57 |
| A chicken Foshan F1025 2018 | 8 | 5 | 7 | 15 | 6 | 2 | 3 | 7 | G57 |
| A chicken Huizhou F169 2017 | 8 | 5 | 7 | 15 | 6 | 2 | 3 | 7 | G57 |
| A chicken Guangzhou F204 2017 | 8 | 5 | 7 | 15 | 6 | 2 | 3 | 7 | G57 |
| A chicken Jiangmen F225 2018 | 8 | 5 | 7 | 15 | 6 | 2 | 3 | 7 | G57 |
| A chicken Yunfu F247 2018 | 8 | 5 | 7 | 15 | 6 | 2 | 3 | 7 | G57 |
| A chicken Shenzhen F433 2018 | 8 | 5 | 7 | 15 | 6 | 2 | 3 | 7 | G57 |
| A chicken Yantai F48 2018 | 8 | 5 | 7 | 15 | 6 | 2 | 3 | 7 | G57 |
| A duck Shenzhen F460 2018 | 8 | 5 | 7 | 15 | 6 | 2 | 3 | 7 | G57 |
| A chicken Guangdong F657 2018 | 8 | 5 | 7 | 15 | 6 | 2 | 3 | 7 | G57 |
| A chicken Dongguan F674 2018 | 8 | 5 | 7 | 15 | 6 | 2 | 3 | 7 | G57 |
| A chicken Shenzhen F65 2018 | 8 | 5 | 7 | 15 | 6 | 2 | 3 | 7 | G57 |
| A chicken Shenzhen F88 2018 | 8 | 5 | 7 | 15 | 6 | 2 | 3 | 7 | G57 |
| A chicken Shenzhen F70 2018 | 8 | 5 | 7 | 15 | 6 | 2 | 3 | 7 | G57 |
| A chicken Yunnan G130 2019 | 8 | 5 | 7 | 15 | 6 | 2 | 3 | 7 | G57 |
| A chicken Qingyuan G220 2019 | 8 | 5 | 7 | 15 | 6 | 2 | 3 | 7 | G57 |
| A duck Qingyuan G264 2019 | 8 | 5 | 7 | 15 | 6 | 2 | 3 | 7 | G57 |
| A chicken Foshan G3 2019 | 8 | 5 | 7 | 15 | 6 | 2 | 3 | 7 | G57 |
| A chicken Guangzhou G41 2019 | 8 | 5 | 7 | 15 | 6 | 2 | 3 | 7 | G57 |
| A chicken Zhanjiang G99 2019 | 8 | 5 | 7 | 15 | 6 | 2 | 3 | 7 | G57 |
