# Supplementary material for: Genetic Evolution Characteristics of Genotype G57 Virus, A Dominant Genotype of H9N2 Avian Influenza Virus
Source: Front Microbiol. 2021 Mar 3;12:633835. doi: 10.3389/fmicb.2021.633835 (PMC7965968; doi:10.3389/fmicb.2021.633835)
Supplement: Supplementary file 4 [file Table_4.docx]

**Table S4** Influenza virus information of H9N2 G57 genotype isolated in China from 2013 to 2017 screened from the NCBI database.

| Isolate | HA | NA |
| --- | --- | --- |
| A_chicken_Tibet_S1_2009 | CY087171 | CY087173 |
| A_chicken_Tibet_S4_2009 | CY087179 | CY087181 |
| A_duck_Tibet_S2_2009 | CY087187 | CY087189 |
| A_duck_Hunan_S4111_2011 | CY146660 | CY146662 |
| A_chicken_Zhejiang_HJ_2007 | FJ581431 | FJ581434 |
| A_chicken_Jiangxi_12_2007 | GQ373076 | GQ373110 |
| A_chicken_Jiangsu_Q3_2010 | JF715006 | JN869532 |
| A_chicken_Zhejiang_Q14_2010 | JF715007 | JN869525 |
| A_chicken_Shandong_01_2010 | JF795038 | JF795040 |
| A_chicken_Shandong_02_2009 | JF795054 | JF795056 |
| A_chicken_Shandong_03_2010 | JF795070 | JF795072 |
| A_chicken_Shandong_BD_2010 | JF795086 | JF795088 |
| A_chicken_Shandong_01_2009 | JF795144 | JF795146 |
| A_chicken_China_AH_10_01_2010 | JF906206 | JF906208 |
| A_chicken_Shuanggou_1_2011 | JN653606 | JN653638 |
| A_chicken_Yongcheng_1_2011 | JN653607 | JN653639 |
| A_chicken_Tongshan_1_2011 | JN653608 | JN653640 |
| A_pigeon_Xuzhou_1_2011 | JN653609 | JN653641 |
| A_chicken_Xigou_1_2011 | JN653610 | JN653642 |
| A_chicken_Jiawang_2_2011 | JN653611 | JN653643 |
| A_chicken_Dawang_1_2011 | JN653612 | JN653644 |
| A_chicken_Qianzhou_12_2010 | JN653613 | JN653645 |
| A_chicken_Anhui_10_2009 | JN653614 | JN653646 |
| A_chicken_Hubei_10_2009 | JN653615 | JN653647 |
| A_chicken_Wuxi_7_2010 | JN653616 | JN653648 |
| A_chicken_Yangzhou_11_2010 | JN653617 | JN653649 |
| A_chicken_Taixing_10_2010 | JN653620 | JN653652 |
| A_chicken_Xiangshui_1_2011 | JN653621 | JN653653 |
| A_chicken_Zhejiang_329_2011 | JQ356872 | JQ356875 |
| A_chicken_Zhejiang_607_2011 | JQ356873 | JQ356876 |
| A_chicken_Zhejiang_611_2011 | JQ356874 | JQ356877 |
| A_chicken_Shanghai_C1_2012 | KC417046 | KC417052 |
| A_chicken_Shanghai_C2_2012 | KC417047 | KC417053 |
| A_chicken_Shanghai_C3_2012 | KC417048 | KC417054 |
| A_brambling_Beijing_16_2012 | KC464598 | KC464600 |
| A_duck_Shanghai_C163_2009 | KC768040 | KC768046 |
| A_duck_Shanghai_C164_2009 | KC768041 | KC768047 |
| A_swine_Henan_Y1_2009 | KC779047 | KC779051 |
| A_swine_Shanghai_Y1_2009 | KC779048 | KC779052 |
| A_chicken_Shandong_sd01_2010 | KC821001 | KC821223 |
| A_chicken_Shandong_06_2010 | KC821002 | KC821227 |
| A_chicken_Shandong_11_2010 | KC821003 | KC821229 |
| A_chicken_Shandong_HL_2010 | KC821004 | KC821233 |
| A_chicken_Beijing_HD_2010 | KC821005 | KC821231 |
| A_chicken_Hebei_fx05_2010 | KC821006 | KC821226 |
| A_chicken_Jiangsu_TS_2010 | KC821007 | KC821234 |
| A_chicken_Shandong_10_2010 | KC821008 | KC821228 |
| A_chicken_Shandong_05_2010 | KC821009 | KC821225 |
| A_chicken_Shandong_sd02_2010 | KC821010 | KC821224 |
| A_chicken_Hebei_YT_2010 | KC821012 | KC821232 |
| A_chicken_Shandong_02_2011 | KC821013 | KC821238 |
| A_chicken_Shandong_05_2011 | KC821015 | KC821243 |
| A_chicken_Hebei_FL_2011 | KC821020 | KC821247 |
| A_chicken_Shandong_06_2011 | KC821021 | KC821244 |
| A_chicken_Shandong_07_2011 | KC821022 | KC821245 |
| A_chicken_Shandong_09_2011 | KC821023 | KC821246 |
| A_chicken_Shandong_11_2011 | KC821024 | KC821248 |
| A_chicken_Sichuan_02_2011 | KC821026 | KC821239 |
| A_chicken_Beijing_HD06_2012 | KF059274 | KF059294 |
| A_chicken_Shandong_zc4_2012 | KF059275 | KF059295 |
| A_chicken_Hunan_12_2011 | KF714783 | KF714785 |
| A_chicken_Gansu_419_2012 | KF715244 | KF715246 |
| A_chicken_Shandong_zc0606_2012 | KM609561 | KM609641 |
| A_chicken_Shandong_yt0106_2012 | KM609563 | KM609643 |
| A_chicken_Shandong_wf1206_2012 | KM609564 | KM609644 |
| A_chicken_Shandong_wf0202_2012 | KM609568 | KM609645 |
| A_chicken_Shandong_qd1224_2012 | KM609569 | KM609648 |
| A_chicken_Shandong_qd1115_2012 | KM609570 | KM609649 |
| A_chicken_Shandong_qd1013_2012 | KM609571 | KM609650 |
| A_chicken_Shandong_qd0920_2012 | KM609572 | KM609651 |
| A_chicken_Shandong_qd0516_2012 | KM609575 | KM609652 |
| A_chicken_Shandong_qd0427_2012 | KM609576 | KM609655 |
| A_chicken_Shandong_lc0830_2012 | KM609583 | KM609656 |
| A_chicken_Liaoning_1116_2012 | KM609586 | KM609663 |
| A_chicken_Liaoning_0704_2012 | KM609587 | KM609666 |
| A_chicken_Jilin_1031_2012 | KM609589 | KM609667 |
| A_chicken_Guangdong_LG1_2013 | KC951122 | KC951124 |
| A_chicken_Tianjin_120_2013 | KF059278 | KF059298 |
| A_chicken_Beijing_11_2013 | KF059281 | KF059301 |
| A_chicken_Wenzhou_253_2013 | KF259160 | KF259562 |
| A_silkie_chicken_Wenzhou_812_2013 | KF259164 | KF259566 |
| A_chicken_Rizhao_853_2013 | KF259173 | KF259576 |
| A_chicken_Rizhao_1313_2013 | KF259174 | KF259577 |
| A_chicken_Shanghai_020_2013 | KF500977 | KF500979 |
| A_chicken_Shandong_yt0711_2013 | KM609562 | KM609642 |
| A_chicken_Shandong_wf0712_2013 | KM609566 | KM609646 |
| A_chicken_Shandong_lc0523_2013 | KM609584 | KM609664 |
| A_chicken_Shaanxi_xa0414_2013 | KM609585 | KM609665 |
| A_chicken_Liaoning_0517_2013 | KM609588 | KM609668 |
| A_chicken_Hebei_0721_2013 | KM609592 | KM609672 |
| A_chicken_Hebei_0109_2013 | KM609593 | KM609673 |
| A_chicken_Beijing_1115_2013 | KM609595 | KM609675 |
| A_chicken_Beijing_0512_2013 | KM609596 | KM609676 |
| A_chicken_Beijing_0309_2013 | KM609599 | KM609679 |
| A_chicken_Jiangsu_NTTZ_2013 | KP657979 |  |
| A_pigeon_Shanghai_JC1_2013 | KJ128362 |  |
| A_chicken_Beijing_0331_2013 | KM609597 |  |
| A_chicken_Shandong_1231_2008 | GQ373072 |  |
| A_chicken_Shandong_SG2_2009 | HM751194 |  |
| A_chicken_Hebei_ZR_2010 | KC821011 |  |
| A_chicken_Shandong_zc12_2009 | KC820999 |  |
| A_swine_Yangzhou_1_2008 | HM998922 | HM998924 |
| A_chicken_Tianjin_614_2012 | KF059277 | KF059297 |
| A_chicken_Shanxi_0703_2012 | KM609560 | KM609640 |
| A_chicken_Shandong_wf12010_2012 |  | KM609645 |
| A_chicken_Shandong_H_2009 |  | JF795096 |
