# Supplementary material for: Genetic Evolution Characteristics of Genotype G57 Virus, A Dominant Genotype of H9N2 Avian Influenza Virus
Source: Front Microbiol. 2021 Mar 3;12:633835. doi: 10.3389/fmicb.2021.633835 (PMC7965968; doi:10.3389/fmicb.2021.633835)
Supplement: Supplementary file 5 [file Table_5.docx]

**Table S5** Positive Darwinian selection of H9N2 G57 genotype in China from 2007 to 2019.

| 2007-2012_HA | Number of positive selection sites | Positive selected site | Non-synonymous/synonymous rate ratio |
| --- | --- | --- | --- |
| FEL | 5 | 3, 4, 13, 145, 198 | 0.225 |
| FUBARD | 5 | 3, 4, 13, 145, 198 |  |
| MEME | 13 | 3, 4, 13, 145, 198, 8, 47, 48, 145, 235, 307, 364, 541, 555, 556, 557 |  |
| 2007-2012_NA | Number of positive selection sites | Positive selected site | Non-synonymous/synonymous rate ratio |
| FEL | 2 | 9, 38 | 0.198 |
| FUBARD | 0 | 0 |  |
| MEME | 4 | 3, 5, 9, 466 |  |

| 2013-2019_HA | Number of positive selection sites | Positive selected site | Non-synonymous/synonymous rate ratio |
| --- | --- | --- | --- |
| FEL | 12 | 4, 5, 13, 17, 66, 75, 87, 88, 145, 168, 198, 353 | 0.178 |
| FUBARD | 8 | 4, 13, 17, 66, 87, 168, 198, 201 |  |
| MEME | 27 | 3,4,5,13,17,66,87,88,145,167,168,196,197,198,205,300,306,307,309,353,497,508,511,512,558,559,560, |  |
| 2013-2019_NA | Number of positive selection sites | Positive selected site | Non-synonymous/synonymous rate ratio |
| FEL | 7 | 3, 8, 31, 127, 208, 266, 369 | 0.214 |
| FUBARD | 3 | 8, 127, 266 |  |
| MEME | 11 | 2, 3, 5, 8, 23, 31, 44, 208, 266, 315, 369 |  |
